# The Inhibition of Interfacial Ice Formation and Stress Accumulation with Zwitterionic Betaine and Trehalose for High-Efficiency Skin Cryopreservation

**DOI:** 10.34133/research.0520

**Published:** 2024-11-14

**Authors:** Xinmeng Liu, Liming Zhang, Haoyue Li, Jing Yang, Lei Zhang

**Affiliations:** ^1^ Department of Biochemical Engineering, Frontier Science Center for Synthetic Biology and Key Laboratory of Systems Bioengineering (MOE), School of Chemical Engineering and Technology, Tianjin University, Tianjin 300350, China.; ^2^ Haihe Laboratory of Sustainable Chemical Transformations, Tianjin 300192, China.

## Abstract

Cryopreservation is a promising technique for the long-term storage of skin. However, the formation of ice crystals during cryopreservation unavoidably damages skin structure and functionality. Currently, the lack of thorough and systematic investigation into the internal mechanisms of skin cryoinjury obstructs the advancement of cryopreservation technology. In this study, we identified 3 primary contributors to skin cryoinjury: interfacial ice nucleation, stress accumulation, and thermal stress escalation. We emphasized the paramount role of interfacial ice nucleation in provoking ice growth within the skin during the cooling process. This progress subsequently leads to stress accumulation within the skin. During the rewarming process, the brittleness of skin, previously subjected to freezing, experienced a marked increase in thermal stress due to ice recrystallization. Based on these insights, we developed a novel zwitterionic betaine-based solution formulation designed for cryopreservation skin. This cryoprotective agent formulation exhibited superior capability in lowering ice nucleation temperatures and inhibiting ice formation at interfaces, while also facilitating the growth of smooth and rounded ice crystals compared to sharp-edged and cornered crystals formed in aqueous solutions. As a result, we successfully achieved prolonged cryopreservation of the skin for at least 6 months, while preserving 98.7% of structural integrity and 94.7% of Young’s modulus. This work provides valuable insights into the mechanisms of ice crystal damage during organ cryopreservation and profoundly impacts the field of organ transplantation and regenerative medicine.

## Introduction

Skin is the largest organ of the body and serves as a crucial protective barrier against environmental factors while providing mechanical support. However, the integrity of this barrier is frequently compromised by various forms of damage, such as thermal burns, traumatic injuries, and pressure ulcers [1–4]. In the United States, around 486,000 patients undergo treatment for skin burns every year, incurring costs of nearly $2 billion [5]. Skin transplantation plays a pivotal role in regenerative medicine, offering vital solutions for severe skin injuries and as a prelusive intervention for complex organ transplants [6–9]. Autologous split-thickness skin grafting is considered the gold-standard treatment for extensive burns and full-thickness skin injuries [10,11]. Nonetheless, due to the scarcity of patients’ skin and the heightened potential for secondary harm, allogeneic skin transplantation has gradually become the preferred treatment option [12,13]. However, the scarcity of donor skin portends a tremendous obstacle to its utilization in clinical practice. A considerable amount of skin grafts is discarded due to inadequate long-term preservation, leading to reduced viability [14,15]. Ex vivo skin grafts may lose up to 50% of their vitality within the first 24 h [16,17]. Therefore, the development of reliable and effective ex vivo skin preservation strategies is urgently needed to mitigate graft injuries.

Cryopreservation is the most effective technology for long-term storage of tissues and organs [18–21]. Many countries have established skin banks by cryopreservation technique including Italy, Israel, India, and Japan. These enable the storage of donor skin grafts in either ultracold −80 °C freezers or liquid nitrogen containers for optimal long-term preservation conditions. However, the freezing and rewarming process can lead to the formation and growth of ice crystals, which pose a significant threat to biomechanical properties and the structural integrity of skin [22–24]. After transplantation, the cryopreserved skin is impaired in terms of sensory nerve regeneration, displaying heterogeneous pigmentation patterns and an absence of hair follicle rejuvenation [1]. Achieving successful preservation of the original physiological functionality and structure integrity of skin remains a challenge [19]. Therefore, a thorough and comprehensive understanding of the mechanisms underlying cryopreservation-induced injuries is crucial for advancing the knowledge of cryoprotective mechanisms and facilitating the rational design of cryoprotective agents (CPAs).

Several theories have been proposed on the dispersed cellular level to explain the formation of intracellular ice crystals, including the pore theory, surface-catalyzed nucleation theory, volume-catalyzed nucleation theory, and cell membrane damage theory [25–28]. However, the mechanisms of ice nucleation and growth during the cryopreservation of multilevel structure skin are currently not fully elucidated [29,30]. Existing research primarily relies on assessing changes in tissue slice estimation, cellular activity, and cytokine levels to indirectly evaluate the mechanisms of cryopreservation damage [31–35]. Further investigation is needed to gain a more comprehensive understanding of these processes. Additionally, the stress generated during freezing, including phase transformation, thermal expansion alterations, and restricted heat transfer, must not be overlooked [36–38]. To address these challenges, some research has been explored including temperature distribution and assessed the implications of thermal stress [32,33,39].

In addition, the utilization of CPAs in skin preservation also presents additional challenges. Glycerol (Gly) (15 wt%) and 15 wt% dimethyl sulfoxide (DMSO) are commonly employed as CPAs for skin and cell cryopreservation [1,16,40–43]. However, it has been observed that DMSO can induce cellular damage and even genetic changes when used at concentrations exceeding 2 wt% [44–47]. Additionally, the limited washout capability of Gly restricts its use in the field of skin preservation [48–50]. Therefore, there is a keen interest among researchers to explore alternative strategies to supplant conventional small-molecule CPAs [19,22]. The zwitterionic betaine has garnered significant attention due to its excellent biocompatibility, potent ice-inhibiting properties, and capacity to regulate osmotic stress. Our research team has conducted comprehensive investigations to assess the efficacy of betaine as a CPA for preserving various cell types, including chondrocytes, red blood cells, GLC-82, and HeLa cells [48,51,52]. However, the elucidation of betaine’s unique ice inhibition mechanism in comparison to other classical permeable CPAs remains a topic that warrants further investigation. Further research is needed to explore its application in organ cryopreservation.

**Fig. 1.**
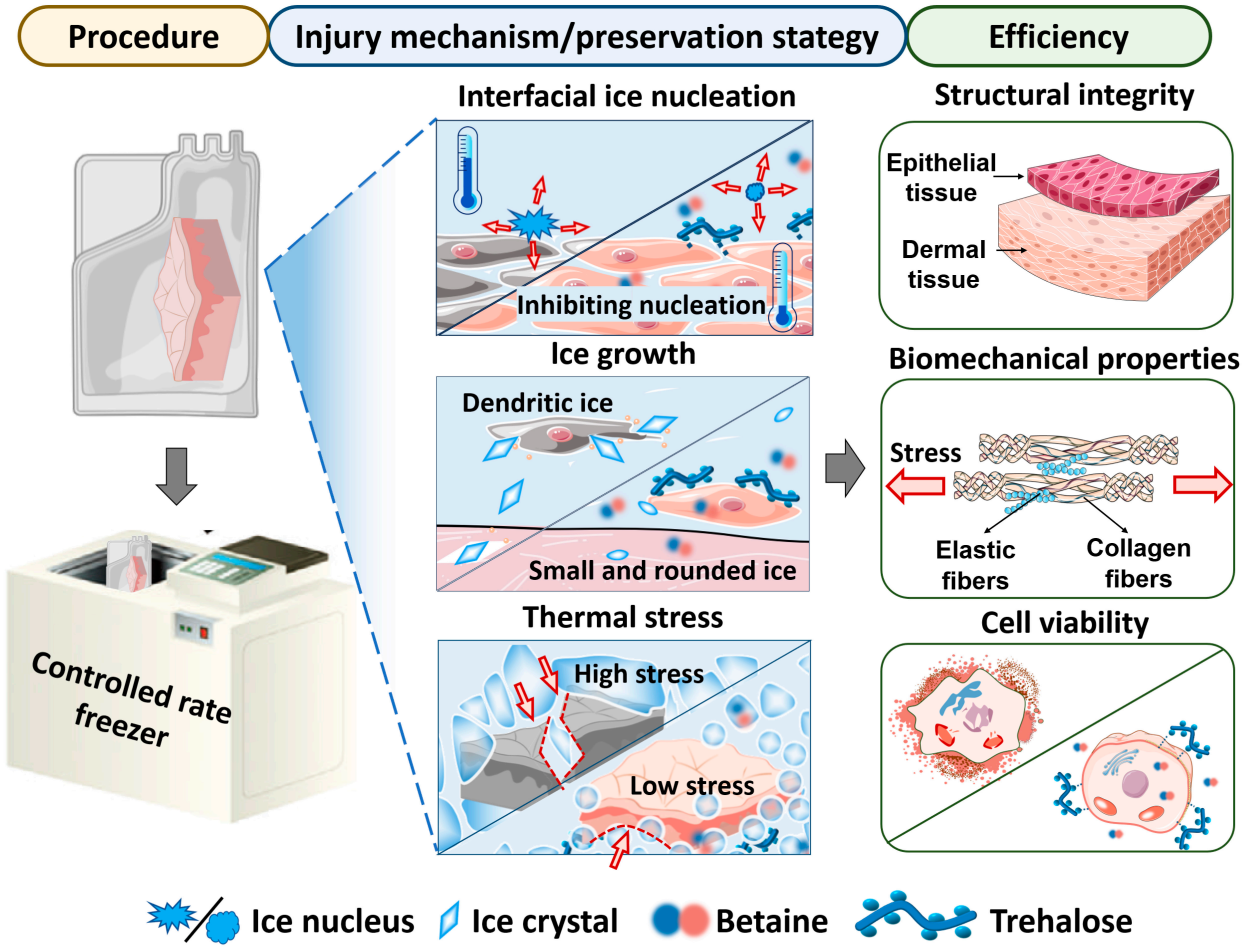
Overview of the injury mechanisms and preservation strategies of skin cryopreservation. The skin cryopreservation procedure involved slow freezing using a controlled rate freezer, followed by storage in a liquid nitrogen tank. During the freezing process, ice nucleation at the liquid–skin interface and subsequent ice growth resulted in damage to the tissue. This progress subsequently leads to stress accumulation within the skin. Upon rewarming, thermal stress exacerbated injury to the frozen skin. The combination of betaine and trehalose (BT) not only reduced the temperature required for ice nucleation but also resulted in smaller and more rounded ice crystals. Additionally, BT reduced the thermal stress by decreasing the thermal expansion coefficient of the frozen skin. The effectiveness of the BT preservation strategy was assessed by evaluating the skin’s structural integrity, biomechanical properties, and viability.

In this study, we focused on exploring the mechanisms of skin injury during cryopreservation and developed a biocompatible protective strategy for long-term preservation (Fig. 1). By combining experimental and computational approaches, we found that (a) ice nucleation occurs primarily at the liquid–skin interface. (b) During the freezing process, changes in phase transition and thermal expansion coefficients result in the accumulation of stress, which can cause damage during long-term storage. Additionally, (c) rapid thermal stress increase during the rewarming process due to phase transition also contributes to skin injury.

To mitigate these cryopreservation-induced damages, we replaced conventional CPAs (such as DMSO and Gly) with excellent biocompatibility zwitterionic betaine formulation for skin cryopreservation. This CPA formulation effectively suppressed ice nucleation and growth while mitigating the stress on the skin, thus improving the stress damage. As a result, the skin exhibited preserved structural integrity and biomechanical properties even after 6 months of cryopreservation.

## Results

### The mechanism of cryopreserved skin injury during cooling process

**Fig. 2.**
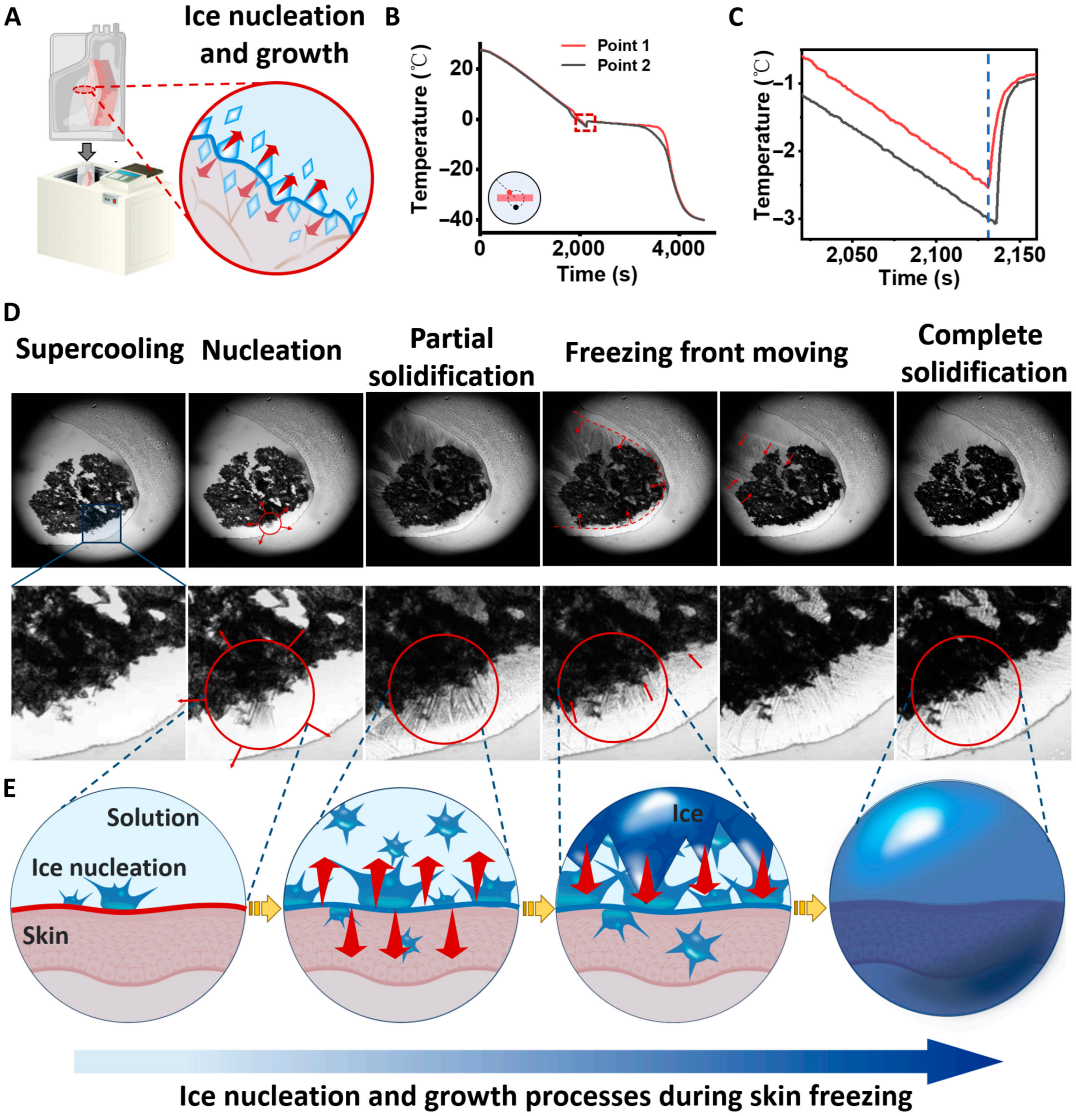
The progress of ice nucleation and growth during skin freezing. (A) Scheme of skin cryopreservation. (B) Temporal variation of temperature with (C) an enlarged detailed view. (D) Microscopic visualization of the skin sample slice with enlarged detailed views. (E) Schematic diagram of ice nucleation and subsequent growth.

In theory, by subjecting the specimens to temperatures below −140 °C, their biological and metabolic activities are significantly slowed down or even halted, inducing a state of “quiescence” or “dormancy” within the organs [18,53]. This state allows the specimens to be preserved for extended periods. However, after cryopreservation of the skin, the biomechanical properties, structural integrity, and viability were irreversibly damaged to prevent utilization [54–56]. To assess the mechanism of cryopreservation injury in the skin during the cooling process, we explored the kinetics of ice nucleation and growth. The skin cryogenic bag filled with solution was frozen at a controlled rate, consistent with clinical strategy (Fig. 2A) [1]. The temperature variation on the skin surface and in external solutions was monitored by a thermal resistance device. To ensure optimal contact between the temperature sensor probe and the skin, we conducted a temperature measurement device as shown in Fig. S1. During the cooling process, the supercooled water was observed due to the high threshold of ice nucleation (Fig. 2B). After ice nucleation, the temperature curve exhibited a slight increase (recalescence), attributed to the absorption of latent heat of fusion by the surrounding solution in the rapid kinetic phase. Subsequently, a gradual thermodynamic phase transition occurred until the freezing process was completed. Notably, focusing on a magnified view in Fig. 2C and Fig. S2, ice nucleation exhibited preferential nucleation at the interface between the skin and the surrounding solution (Point 1 manifested prior to Point 2). As for the rapid cryopreservation system, the same phenomenon has been documented when using plunging cooling in liquid nitrogen (Fig. S3). Besides, to assess freezing behavior within the skin during cryopreservation, we monitored the temperature variation inside the skin and in surrounding solutions as shown in Fig. S4. Compared to the interior of the skin, ice nucleation and the completion of water freezing in the surrounding solution occurred significantly earlier.

To further verify preferential ice nucleation at the liquid–skin interface during the freezing process, we performed a visual experiment by a high-speed imaging microscope system. The skin sample slice was immersed in a thin layer of aqueous solution with cryostage cooling (Fig. 2D). The results demonstrated the formation of ice nucleation (gray value reduction in red circle) at the liquid–skin interface. Subsequently, dendritic crystal rapidly propagated with the initiation of the ice front at the liquid–skin interface and extended upwards until complete solidification. Besides, the scheme of the ice nucleation and growth process was elucidated in Fig. 2E. In a word, due to the combined effects of ice nucleation at the liquid–skin interface and dendritic growth of ice crystals, the skin may be susceptible to damage during the freezing process.

**Fig. 3.**
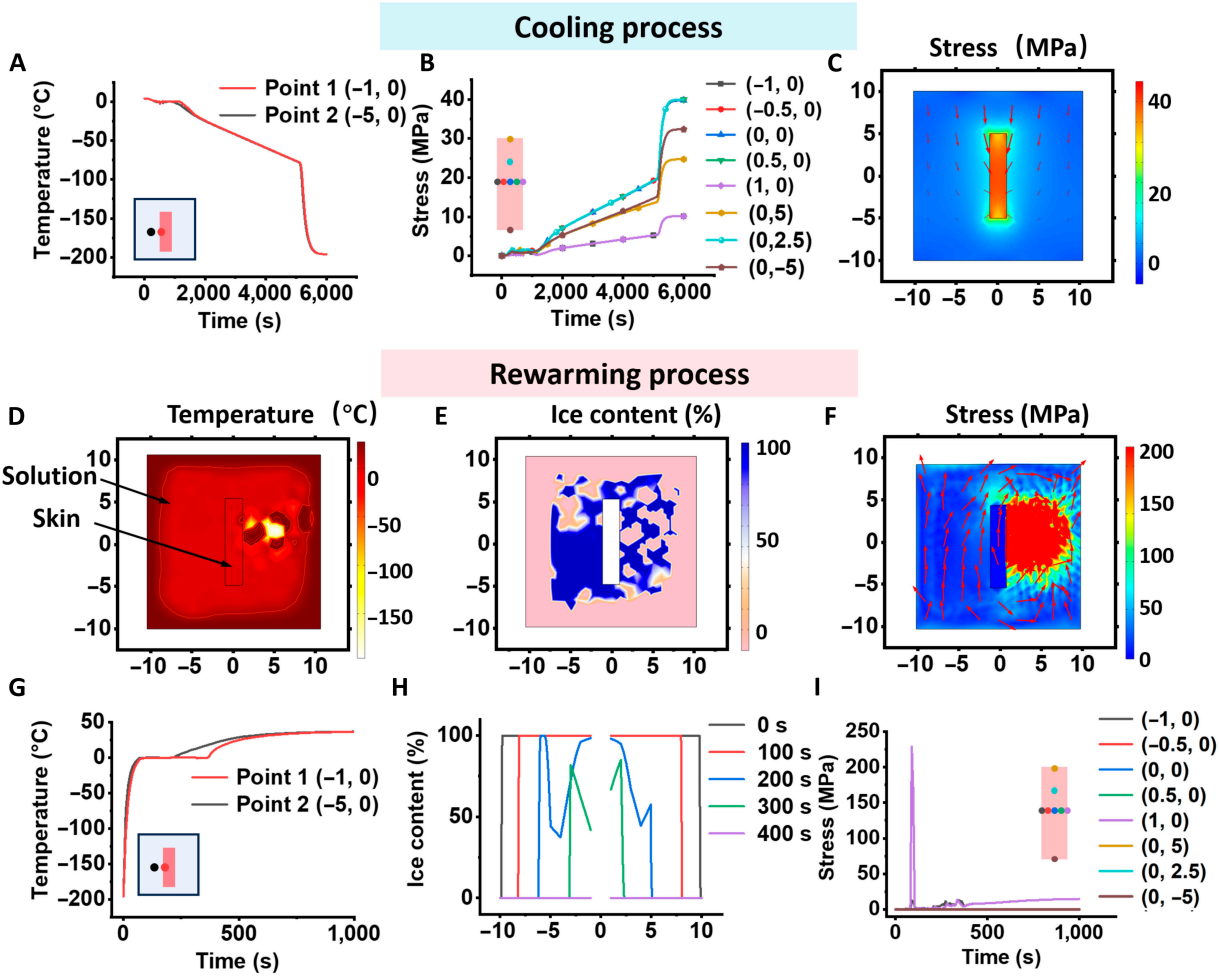
The finite element analysis of the cooling and rewarming process on skin cryopreservation. During the cooling process, (A) the temperature distribution of the interface between the skin and the surrounding aqueous solution, (B) stress distribution with different skin regions, and (C) simulation snapshots of the cryopreserved skin in the LN2 at the time of 6,000 s. During the rewarming process, simulation snapshots of (D) temperature, (E) ice content, and (F) stress at the same time for 88 s. The red arrow represents the stress direction. (G) Temperature variation of the skin in contact (Point 1) and the skin situated at a certain distance (Point 2, same concentric circles of Point 1). (H) Phase transition behavior. (I) Thermal stress distribution with different skin regions.

To assess the stress during the freezing process of the skin, we used COMSOL Multiphysics software to establish a finite element model . The system setup involved immersing the skin in the aqueous solution with a controlled cooling rate applied at the boundary. The temperature was gradually decreased at a rate of −1 °C/min until it reached −80 °C, followed by cryopreservation in liquid nitrogen. The simulation parameters were shown in Table S1. Figure S5A and B illustrate the simulation of the water/ice phase transition. The initiation of ice formation at the boundary of the box (*x* = 10 mm, *x* = −10 mm) at the time of 350 s, attributed to the boundary acting as a cold source where heat gradually transfers inward. Over time, the ice progressively extended toward the interior, eventually leading to full freezing at the 1,300 s. The temperature distribution obtained from the model in Fig. 3A was consistent well with the experimental data. The distribution of internal skin temperature and stress were simulated in Fig. S5C to F. These indicated that significant stress on the skin was caused by the phase transition of the surrounding solution. Attributed to the phase and the thermal expansion between the surrounding aqueous solution and the skin, Fig. 3B and C reveal a significant increase in stress. Besides, the higher stress experienced inside the skin compared to its boundary may be attributed to the accumulation of stress within the skin, possibly arising from the different thermal expansion coefficients between skin and the external solution surrounding it. Noteworthily, the accumulation of stress during cryopreservation could cause damage to the skin. To substantiate this concept, we evaluated the structural integrity, viability, and biomechanical properties in Figs. S6 to S8. The results illustrated a progressive exacerbation of skin damage with extended cryopreserved periods.

### The mechanism of cryopreserved skin injury during the rewarming process

The temperature distribution of the interface between the skin (Point 1) and the surrounding aqueous solution (Point 2) was tested by a water bath rewarming method (Fig. S9). An observable temperature plateau indicated the commencement of a phase transition to water, which persisted until the transition was completed. Notably, the completion of phase transition at point 1 lagged behind that at point 2 in the process of temperature transfer from the external to the internal environment of the system. This is due to the relatively low heat transfer coefficient of the skin.

Through finite element analysis, we extended the temperature range from −196 to 37 °C to investigate temperature variation, phase transition, and thermal stress distribution in the system (Fig. 3D to F). Analysis of the temperature variation depicted in Fig. 3G and Fig. S10 exhibited a trend in line with the experimental findings and affirmed the reliability of the simulation. Besides, the phase transition behavior and thermal stress distribution were further explored in Fig. 3H and I. Due to heat provided at the boundaries, ice content increased gradually from the edges toward the interior, melting completely by 400 s. During the rewarming process, the phase transition of ice melting induced tensile stress on the skin. Concurrently, the skin expansion occurred as a consequence of increased temperature, resulting in a culmination of tensile stress reaching up to 228.7 MPa in the vicinity of the skin boundary (at the time of 88 s). Besides, the higher stress along the x-axis direction of the skin compared to the y-axis direction can be attributed to the longitudinal stretching effect experienced by the skin during the rewarming process.

In comparison to the freezing process, the skin experienced greater stress during the rewarming phase. During the cooling process, the skin retained elasticity despite experiencing compressive stress from the water phase transition, while the skin remained brittle due to freezing during the rewarming process. Thus, the skin became more prone to heightened stress and increased susceptibility to severe injury during the rewarming process. The preservation of skin during the pivotal stage of rewarming following cryopreservation constitutes an imperative priority.

### The ice inhibition and thermal expansion of CPAs

**Fig. 4.**
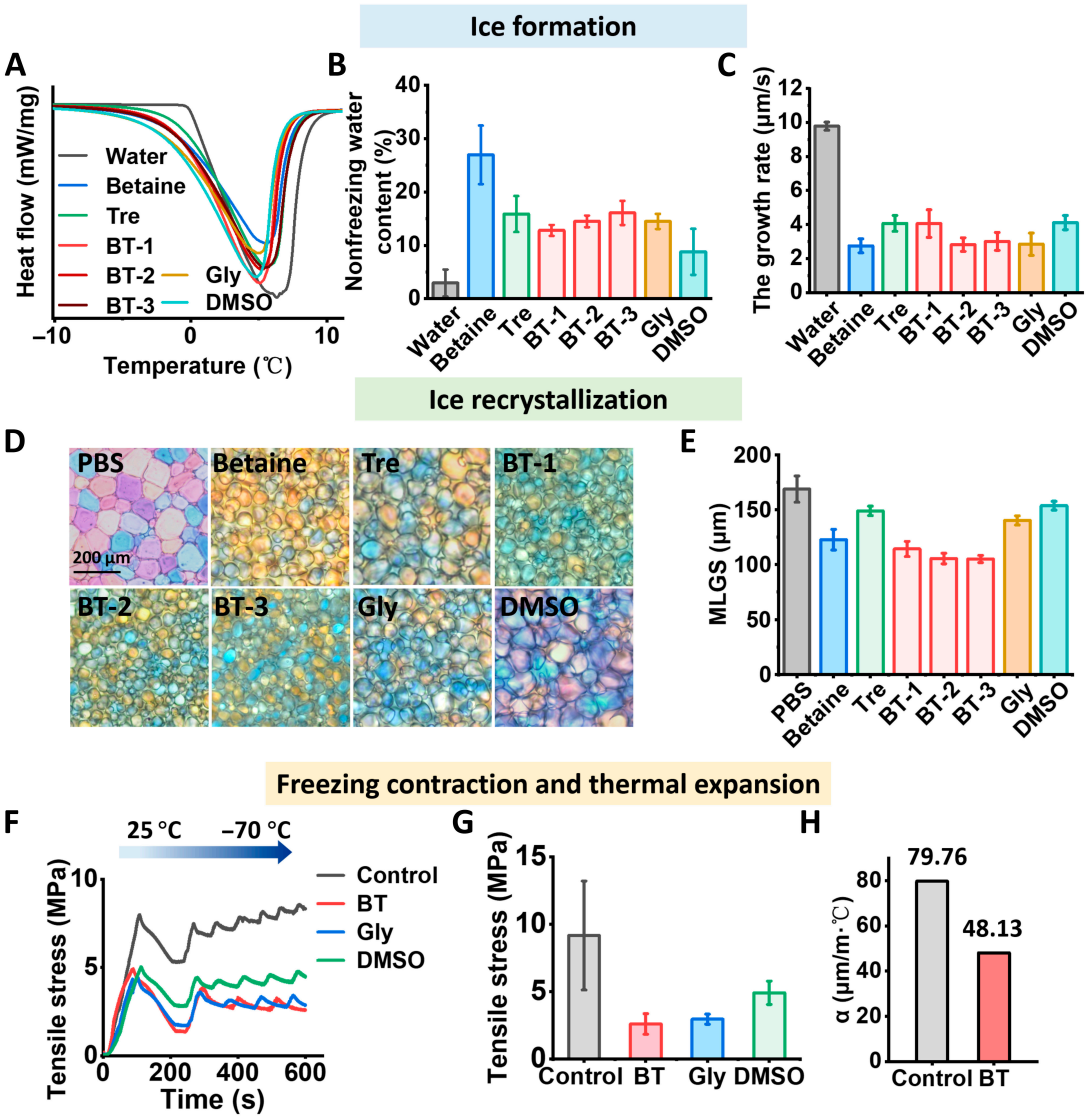
The ice inhibition and thermal expansion of CPAs. (A) Differential scanning calorimetry thermograms. BT-1: 8 wt% B + 7 wt% T; BT-2: 10 wt% B + 5 wt% T; BT-3: 12 wt% B + 3 wt% T. (B) Nonfreezing water contents. (C) The quantifiable data of ice growth rates of different CPAs. (D) Cryomicrographs and (E) quantitative assessment of ice recrystallization inhibition effect. (F) Tensile stress with the temperature range of 25 and −70 °C. (G) The tensile stress is at −70 °C. (H) Thermal expansion coefficient (α) of skin with BT and without CPAs (control group).

Ice formation and growth pose substantial challenges to the preservation of skin integrity and functionality during the cryopreservation processes [19,57]. Thus, the control and inhibition of ice formation are a critical aspect in mitigating injuries during skin cryopreservation. We investigated the impact of CPAs on skin cryopreservation with a focus on ice nucleation and growth behavior (including size, shape, and growth rate). To evaluate the influence of betaine and trehalose (BT) solution on ice formation properties, the differential scanning calorimetry analysis was performed in Fig. 4A. During the cooling process of CPAs, a part of the water forms ice, while another part remains in an unfrozen state, referred to as nonfrozen water (more details are provided in the Supplementary Materials). The obtained thermographs revealed that all CPAs exhibited melting peaks. Notably, the betaine group demonstrated a smaller melting peak area, lower freezing point (−1.58 °C), and increased nonfreezing water content (27.0%) compared to both conventional CPA (Gly and DMSO) groups and water group (Fig. 4B and Fig. S11). These observations highlight the outstanding ability of the betaine group to suppress ice formation. Notably, the betaine solution exhibited the excellent ability of ice nucleation inhibition (Fig. S12). Besides, ice nucleation triggered nearby water molecules to quickly arrange themselves in a regular pattern, which leads to rapid ice growth [58–61]. To assess the impact of the BT solution on inhibiting ice growth, we measured the growth rate of individual ice crystals as shown in Fig. 4C and Fig. S13. The results demonstrated that the betaine group effectively inhibited ice crystal growth (2.75 μm/s) compared to the aqueous solution (9.79 μm/s). BT solution also exhibited a better inhibitory capacity of ice growth, particularly the BT-2 solution (2.81 μm/s). Moreover, compared to the water group where ice growth appeared dendritic, the ice front in the BT solution displayed a smoother and more rounded morphology (Fig. S14). Therefore, during the cooling process, BT solution has the remarkable ability to inhibit ice nucleation and growth.

The phenomenon of ice recrystallization during the rewarming process demands attention. The undesirable growth of larger ice crystals at the expense of smaller ones, known as Ostwald ripening, exacerbates stress levels experienced by biological samples [52,62]. To address this issue, we evaluated the ice recrystallization ability of BT solution by splat cooling assay (Fig. 4D and E). The inclusion of BT molecules effectively inhibited the ice recrystallization process. Specifically, a remarkable difference in BT was found in the morphology of the ice crystals formed with the smooth and round crystals compared with the sharp-edged and cornered crystals seen in the control group. Given the parameters of concentration and ice inhibition efficacy, the BT-2 solution (subsequently denoted as BT) has been selected as the subject for our subsequent research.

It is crucial to acknowledge the potential deleterious impact of the abrupt surge in thermal stress on the integrity of biological samples during the rewarming process [56,63]. Based on the above discussion, significant stress in the skin was conducted due to the phase transformation and the fragility of the skin caused by freezing during the rewarming process. We investigated the influence of the BT solution on the freezing contraction and thermal stress behavior of the skin. The skin samples without CPAs (control group) experienced considerable stress during the freezing process, whereas the presence of BT significantly reduced stress levels (Fig. 4F and G). This beneficial effect of BT could be attributed to its ability to improve the thermal expansion behavior of the skin. Experimental evidence showed that the incorporation of BT resulted in a remarkable 40% decline in the skin’s thermal expansion coefficient, surpassing that of the skin without CPAs. (Fig. 4H and Fig. S15). Consequently, skin protected with BT demonstrated enhanced elasticity in the frozen state and effectively endured higher stress levels (Fig. S16). The BT solution demonstrates considerable promise for skin cryopreservation.

### The molecular-level cryopreservation mechanism of CPAs

**Fig. 5.**
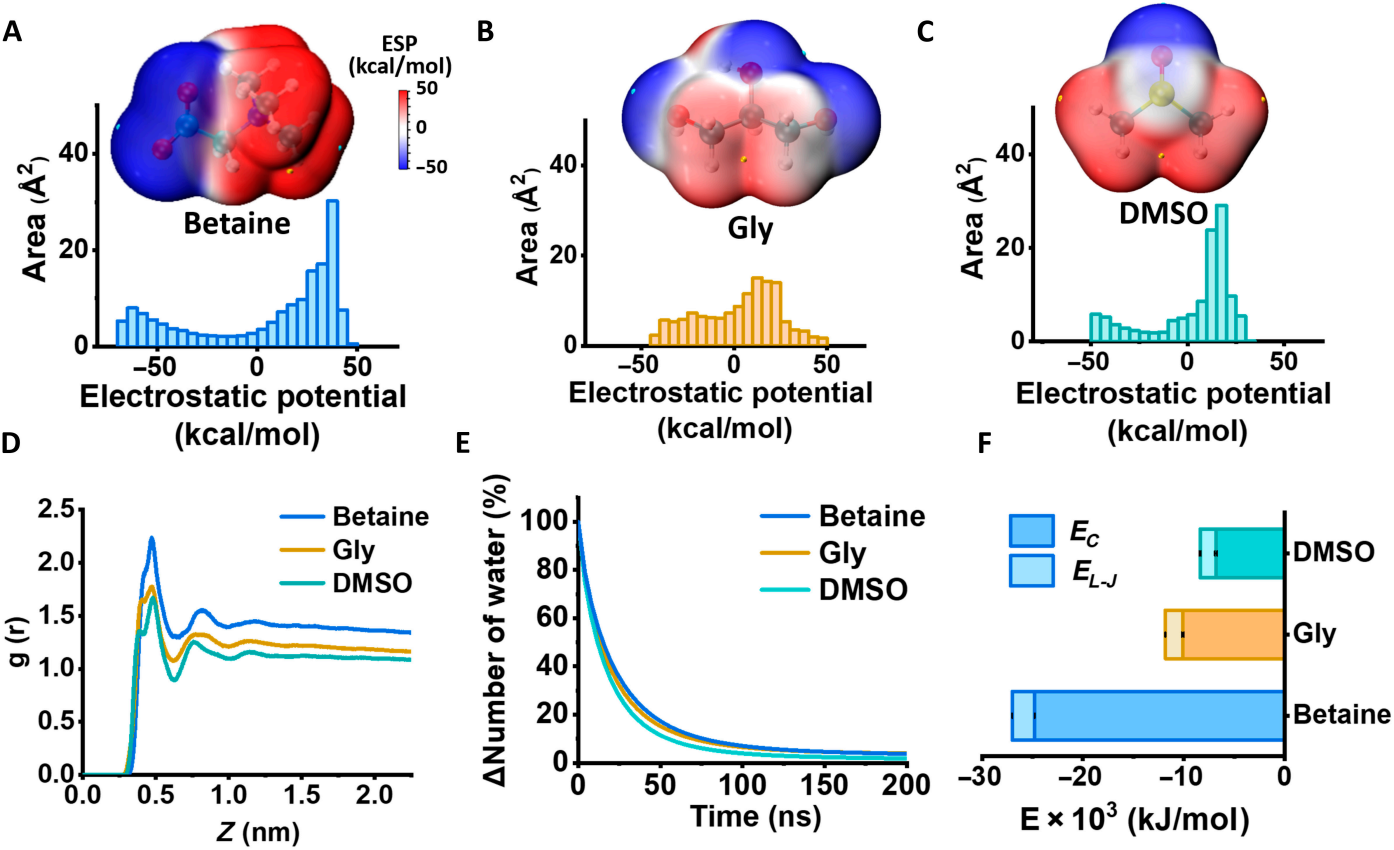
MD simulation of the molecular-level cryopreservation mechanisms of CPAs. ESP distribution and DFT-calculated structural snapshots of (A) betaine, (B) Gly, and (C) DMSO molecules. (D) The radial distribution function g(r). (E) Residence times of the water molecules at the first solvation shell. (F) Interaction energy with water molecules, including electrostatic interaction (*E_C_*) and van der Waals interaction (*E_L-J_*).

The effectiveness of CPAs in inhibiting ice formation and managing thermal expansion relies heavily on the interactions between the CPAs and water/ice molecules. In pursuit of a comprehensive comprehension of the cryopreservation mechanism of permeable CPAs (betaine, Gly, and DMSO), molecular-level simulations were implemented to elucidate the role of CPA molecules within the ice–water system. Molecular surface analysis was employed to quantitatively evaluate the noncovalent interaction of CPA molecules. Specifically, electrostatic potential (ESP) distribution maps of betaine, Gly, and DMSO molecules were demonstrated in Fig. 5A to C and Fig. S17. The ESP of molecular betaine displayed its lowest negative values in close proximity to the 3 oxygen atoms, while the highest positive values were observed near the hydrogen atoms bonded to nitrogen and oxygen. This signified a heightened propensity for binding with water molecules compared to conventional permeable CPAs (Gly and DMSO) molecules. Subsequently, molecular dynamics (MD) simulations were employed to assess the interaction between these CPA molecules and water molecules at the molecular level during the freezing process. Figure 5D shows the radial distribution functions of CPA molecules’ surface, which describes the density of water molecules as a function of distance from CPAs. The results indicated that the first solvation shell of molecular betaine appeared at 4.7 Å. The density and the residence times of water molecules around molecule betaine were notably higher compared to molecules Gly and DMSO at the first solvation shell (Fig. 5E). Besides, the interaction between water molecules and CPA molecules was analyzed in the freezing process. The unique amphipathic nature of betaine molecular exhibited robust interaction energy with water molecules, primarily driven by electrostatic interactions (Fig. 5G). The disruption of the regular alignment of water molecules is attributed to the interaction, leading to the increased formation of unfrozen water, corroborating the findings discussed earlier. The results indicated that betaine molecules exerted a significant effect on the cryopreservation of skin, which has the potential to replace conventional permeable CPAs.

### Histology and viability of skin following cryopreservation

**Fig. 6.**
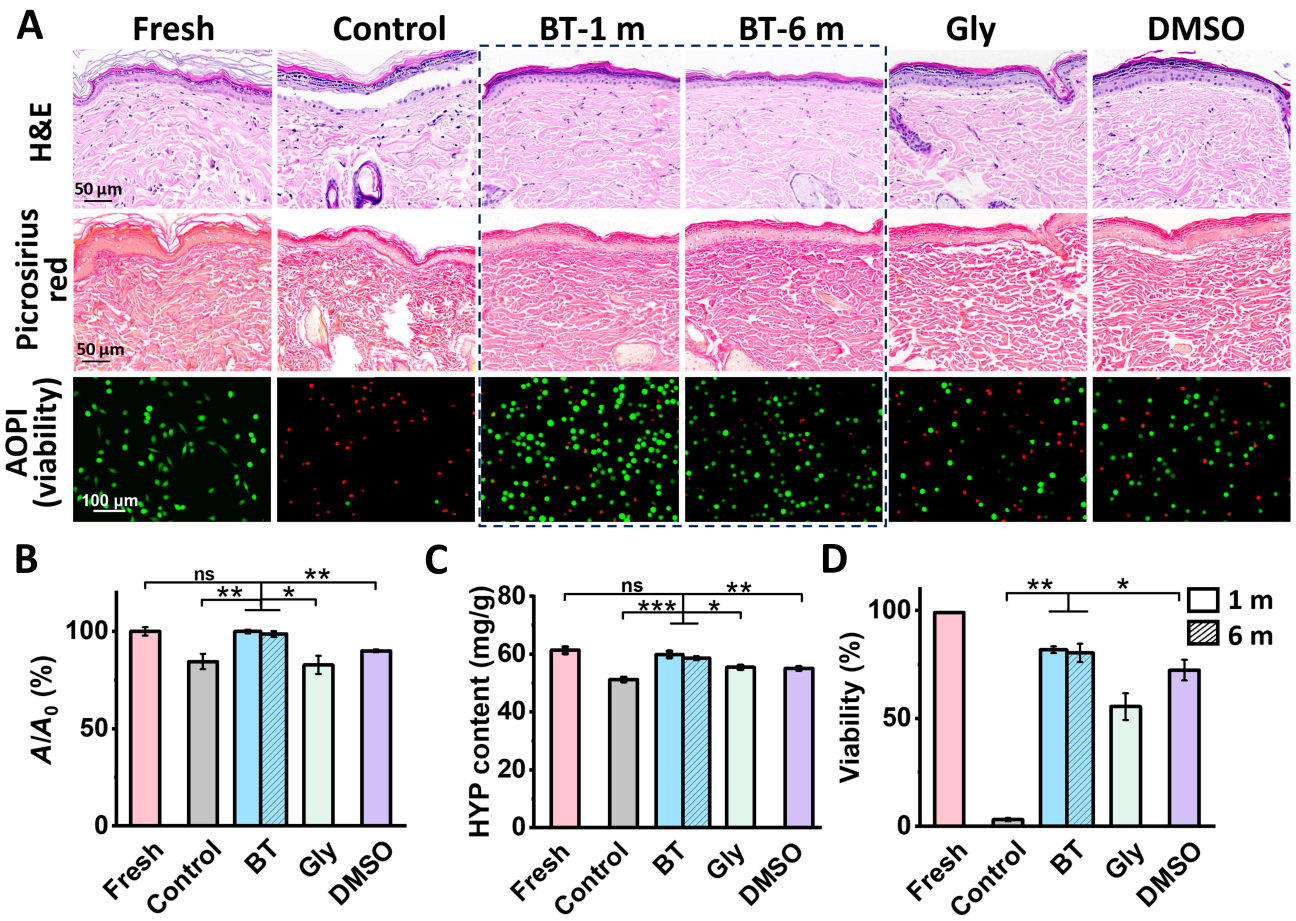
Histology and viability of skin following cryopreservation. (A) Micrographs of fresh skin and cryopreserved skin stained with H&E (purple: chromatin within the cell nucleus and nucleic acids; eosinophilic pink: the cytoplasm and extracellular matrix), Picrosirius Red (red: collagen fibers; yellow: muscle bundles), and live/dead staining (green: live cells; red: dead cells). (B) Skin tissue density quantification. (C) HYP content. (D) Cell viability. Preservation times were 1 month for groups Control, Gly, DMSO, and BT, extending to 6 months for BT. Control group: cryopreserved skin without CPAs.

To evaluate the protective efficacy of the BT formulation, we employed the optimized duration of BT penetration and gradient freezing rate were employed for skin cryopreservation (Figs. S18 and S19). The structural integrity and collagen fiber content of cryopreserved skin samples were assessed by hematoxylin and eosin (H&E) staining kit, Picrosirius Red staining kit and hydroxyproline (HYP) analysis kit. The results of H&E staining indicated that the BT group maintained dense tissue with relatively uniform cell nuclear morphology post cryopreservation, for both 1 and 6 months of cryopreservation (Fig. 6A). Remarkably, structural integrity was maintained at 98.7% even after extending the cryopreservation duration to 6 months (Fig. 6B). In contrast, even after 1-month cryopreservation, nonuniform cell nuclear morphology was observed in the Gly group and was even more pronounced in the DMSO group, possibly due to the toxicity of this agent [64]. Given that collagen protein constitutes approximately 70% of the dermal matrix and plays a crucial role in maintaining skin structural integrity, Picrosirius Red staining was performed to analyze collagen fiber arrangement and distribution in the cryopreserved skin [65,66]. After 6 months of cryopreservation, the BT group exhibited collagen fiber arrangement and distribution comparable to the fresh group. Moreover, the quantitative assessment of collagen content using the HYP assay indicated no significant difference between the BT and fresh groups, thus further validating the positive impact of BT on skin structural integrity during the freezing process (Fig. 6C).

The vitality of cryopreserved skin is crucial for maintaining the structural integrity of skin tissue and supporting the survival of allografts [16,67]. The presence of viable cells promotes the formation of blood vessels in skin grafts, aiding their integration at the site of injury [68,69]. H&E staining images demonstrated preserved nuclear morphology in the BT group following cryopreservation. To evaluate the effects of BT solution on cell viability of ex vivo skin, we used a collagenase enzyme digestion method to extract the skin cells. These extracted cells were mainly fibroblasts along with a minor population of keratinocytes [70]. The skin cells without CPAs (control group) resulted in a marked decrease in viability for only 1 month of cryopreservation, primarily due to the formation of sharp ice crystals causing mechanical damage and the consequent cell dehydration resulting from increased extracellular osmotic pressure during the freeze–thaw process. However, when the BT formulation was applied, the viability of cryopreserved cells after 1 and 6 months increased to 81.8% and 80.3%, respectively (Fig. 6D). These may contribute that the BT group effectively regulated intracellular and extracellular osmotic pressure and facilitated the formation of smaller and smoother ice crystals, thereby minimizing freeze–thaw-induced damage. Notably, BT provided a longer window of cell viability during the rewarming process, compared to conventional CPAs. The superior protective capabilities of BT can be attributed to its unique chemical composition, which enhances its ability to regulate osmotic pressure and minimize freeze–thaw-induced damage, making it a promising candidate for skin cryopreservation.

### Biomechanical properties of skin following cryopreservation

**Fig. 7.**
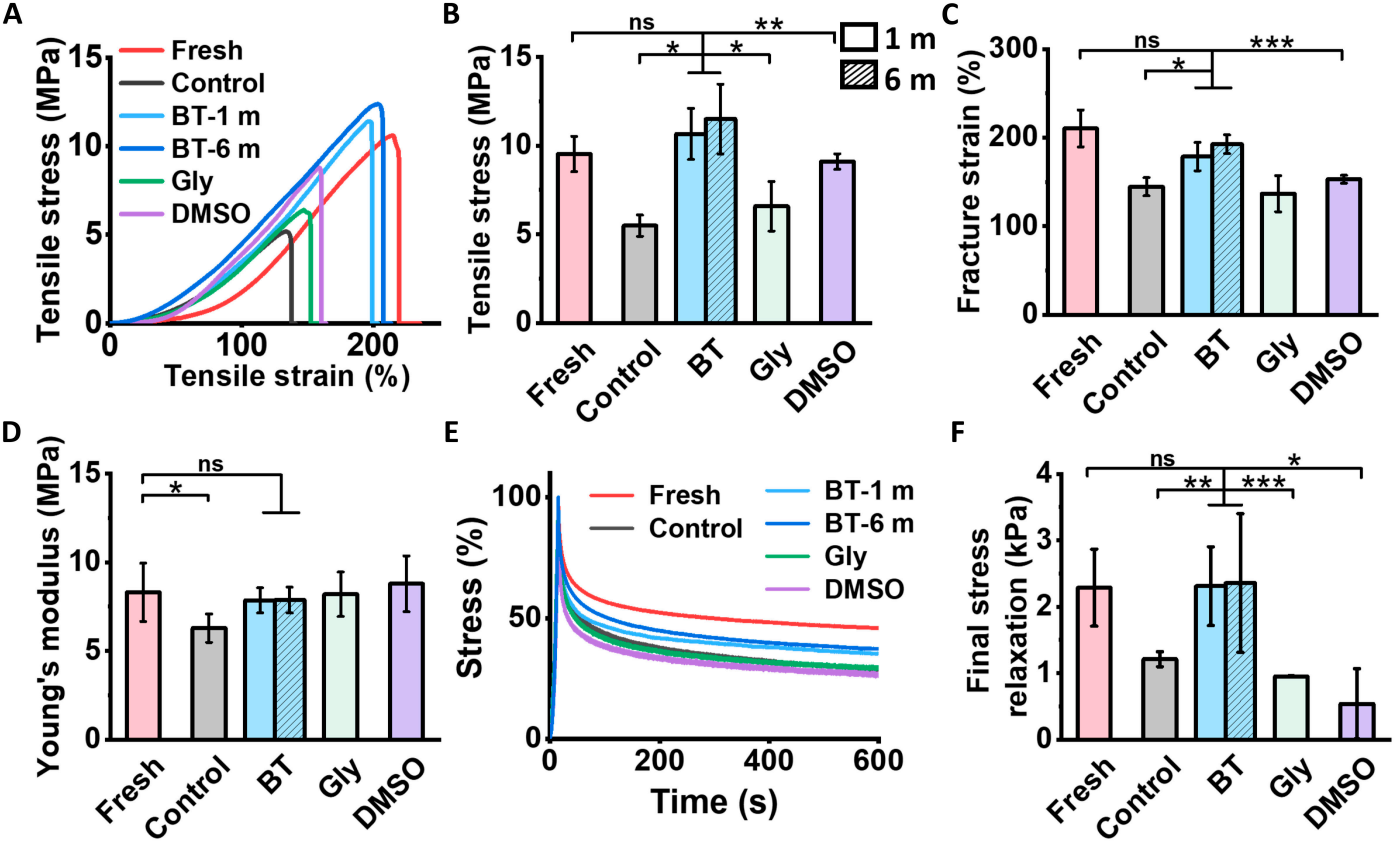
Biomechanical properties of skin following cryopreservation. (A) Stress–strain curve. (B) Maximum tensile stress. (C) Fracture strain. (D) Young’s modulus. (E) Stress relaxation curve at 25% strain. (F) Final stress relaxation. One month of cryopreservation: Control, Gly, DMSO, and BT groups. Six months of cryopreservation: BT group. Control group: cryopreserved skin without CPAs.

The skin plays a vital role in protecting the body from external forces and potential harm. To assess the biomechanical properties of cryopreserved skin, the tensile stress–strain responses of skin were established. The stress–strain curve displayed a distinctive “J” shape and 3 regions (I-toe region, II-heel region, and III-linear region), primarily attributed to collagen fibers and elastic fibers (Fig. 7A and Fig. S20) [71–73]. Initially (region I), the elasticity properties of the skin were primarily by delicate elastic fibers, while the collagen fibers displayed coiled conformation [74]. With increasing strain (region II), the collagen fibers gradually were elongated, leading to a progressively steeper slope in the curve [75,76]. In region III, the collagen fibers exhibited complete elongation reached their maximum extension and served as the predominant load-bearing structures. According to the stress–strain curve, the maximum stress, fracture strain, and Young’s modulus of cryopreservation skin with/without CPAs were evaluated (Fig. 7B to D). After 1 month of cryopreservation, the control group (without CPAs) significantly compromised the maximum stress, fracture strain, and Young’s modulus of the skin. This may be attributed to the formation of ice crystals, which damaged the collagen proteins and reduced collagen content, as observed in HYP analysis. In contrast, the cryopreserved skin with BT showed no significant differences in maximum tensile stress and fracture elongation compared with fresh skin for both 1 and 6 months of cryopreservation. Besides, the BT group outperformed conventional CPAs (Gly and DMSO groups) due to the formation of small and rounded ice crystals.

Viscoelasticity is a prominent characteristic of skin’s mechanical properties, which were evaluated by stress relaxation behaviors [77,78]. In the I-zone, where elastic fibers were predominantly stretched, the curling of collagen fibers primarily contributed to viscosity. The III-zone, where the collagen fibers are fully extended, primarily served as the primary structure for elasticity, and inter-fiber slip primarily contributed to viscosity. Stress relaxation behavior of the skin was tested at fixed strains of 25% (I-zone) and 100% (III-zone) in Fig. 7E and F and Fig. S21. The results indicated that the BT group, even after 6 months of cryopreservation, exhibited significantly enhanced residual elasticity and stress relaxation compared to the control group cryopreserved for merely 1 month. The viscoelasticity of the BT group surpassed that of the conventional CPAs group, indicating the excellent preserve capacity of the BT to and sustain the viscoelastic properties of cryopreserved skin.

## Discussion

The transplantation of large, viable, and functional skin grafts to patients with extensive skin defects has been shown to effectively enhance wound healing and alleviate patient pain. However, the success of transplantation is often hindered by the limited availability of high-quality skin grafts, exacerbated by constraints in preservation methods. In order to address this challenge, our research focused on understanding the mechanisms behind cryopreservation-induced damage to skin. We summarized 3 conclusions: (a) ice nucleation occurs primarily at the liquid–skin interface. (b) Stress accumulation impacts the long-term cryopreservation during the freezing process. (c) The abrupt amplification of thermal stress results in heightened susceptibility of the brittle skin to injury during the rewarming process. Based on these insights, we developed a betaine-based CPA formulation for skin cryopreservation. This CPA formulation effectively suppressed ice nucleation and growth while mitigating the stress on the skin, leading to preserved structural integrity and biomechanical properties of the skin after 6 months of cryopreservation. A comprehensive understanding of the underlying mechanisms of cryopreservation-induced skin tissue damage will enable us to devise effective strategies to mitigate such damage, thereby enhancing the availability of high-quality grafts for transplantation.

## Materials and Methods

### Rats skin procurement

Male Sprague-Dawley rats, aged 10 weeks and weighing between 300 and 320 g, were procured from Beijing Huafukang Bioscience Co. Ltd., Beijing, China. Our study was conducted in accordance with animal welfare guidelines and received ethical approval from the Committee of the Laboratory Animal Science Department at the Institute of Radiation Medicine.

### Cooling and warming procedure

To prepare the CPAs, individual solutions were prepared by phosphate-buffered saline (PBS) solution with the following compositions: 15 wt% betaine, 15 wt% Tre, 15 wt% BT (BT-1: 8 wt% betaine + 7 wt% Tre; BT-2: 10 wt% betaine + 5 wt% Tre; BT-3: 12 wt% betaine + 3 wt% Tre), 15 wt% Gly (common CPA for skin cryopreservation), and 15 wt% DMSO (common CPA for skin cryopreservation). The ex vivo skin samples were immersed in these CPAs using cryogenic Ziplock bags at a temperature of 4 °C for a period of 2 h. A controlled-rate freezer (SY-Lab, Austria) was employed to gradually lower the temperature of the skin samples at a controlled rate of −1 °C/min until reaching a temperature of −80 °C. Subsequently, the frozen skin samples were transferred to liquid nitrogen cryopreservation.

For the rewarming process, the cryopreserved skin samples were rapidly rewarmed by immersing the cryogenic Ziplock bags in a water bath heated to 37 °C for a period of approximately 2 to 3 min, or until complete ice melting occurred. Post rewarming, the skin was subjected to a meticulous purification procedure involving continuous rinsing with flowing water to eliminate any residual CPA. Subsequently, the purified skin was immersed in a physiologically balanced saline solution at a temperature of 4 °C, ensuring its suitability for further application.

### Temperature and stress distribution simulation

The thermal and stress distribution of skin during the cooling and rewarming process were evaluated by COMSOL Multiphysics 5.6. To study the cryopreservation of skin tissues in a cryovial submerged in liquid nitrogen, a 2-dimensional model was developed. The model comprises a large square (20 mm × 20 mm) representing the surrounding water environment and a rectangular shape depicting the skin tissue sample (10 mm × 2 mm) placed within it. The skin and the surrounding aqueous solution were described as materials with linear elasticity and isotropic properties. The simulation parameters were provided in the Supplementary Materials.

In the solid mechanics model, the total strain rate in the skin and surrounding aqueous solution model is determined using the Maxwell fluid model as follows:

ε˙=ε˙elastic+ε˙creep+ε˙thermalε˙elastic=1E1+vσ˙−vΙ·trσ˙ε˙creep=S2με˙thermal=αT˙Ι (1)

where the formulation involves the stress tensor ($\dot{\sigma }$), deviatoric stress tensor (*S*), Young’s modulus (*E*), viscosity (*μ*), as well as the elastic (${\dot{\varepsilon }}_{\text{elastic}}$), creep (${\dot{\varepsilon }}_{\text{creep}}$), and thermal (${\dot{\varepsilon }}_{\text{thermal}}$) stress tensors. Additionally, it incorporates the Poisson’s ratio (*v*), identity matrix (***Ι***, the bold symbols employed signify vector fields), trace of a matrix (*tr*), and linear thermal expansion coefficient (*α*).

To address the volumetric variations arising from the freezing and thawing of the water within the cryovial, roller-supported boundary conditions are imposed on the lateral walls of the cryovial. Furthermore, a fixed boundary condition is enforced at the base of the cryovial, while the upper boundary is treated as free.

In the fluid and solid heat transfer model, the governing equation for the coupling of heat transfer and fluid flow through natural convection is given as follows:

dZρCP∂T∂t+dZρCPu·∇T+∇·q=dZQ+q0q=−dZk∇T (2)

where *ρ* represents density, *d_Z_* is system thickness, *C_P_* is specific heat, T is temperature, t is time, ***u*** is velocity field, *Q* is heat absorbed by the system, *q*_0_ is initial heat flux, ***q*** is heat conduction flux vector, and *k* is the thermal conductivity.

Due to the phase transition between water and ice of the skin surrounding, phase-change materials were described in fluid model.

ρ=θ1ρ1+θ2ρ2CP=1ρθ1ρ1CP,1+θ2ρ2CP,2+L1→2∂αm∂Tαm=12θ2ρ2−θ1ρ1θ1ρ1+θ2ρ2k=θ1k1+θ2k2θ1+θ2=1 (3)

where the subscripts 1 and 2 indicate the materials before (phase 1) and after (phase 2) the phase transition. *θ* represents the percentage of a certain phase, and *α_m_* is the thermal expansion coefficient.

### The permeation kinetics of CPAs in skin

In order to determine the optimal loading time of CPAs, a study was carried out on the kinetics of skin permeation. The objective was to achieve equilibrium permeation of the CPA from the skin into PBS solution. The permeation of the surrounding solution adjacent to the skin was measured using a freezing point osmometer (Tianda Tianfa, China), enabling the evaluation of CPA penetration into the skin. The specific calculation method employed is as follows [79,80]:

nCPA=πCPA−πPBS∗VPBS∗ρWWtCPA=nCPA∗MWcpaVCPA=WtCPA/ρcpaCCPA=nCPAVCPA+W2−W1−WtCPAρ (4)

Following immersion in CPA for varying durations (ranging from 1 to 180 min) at 4 °C, the skin, along with the immersed CPA, was subsequently placed in PBS until osmotic pressure equilibrium was achieved (*π_CPA_*). Any CPA residue left within the skin was not taken into account. *π_PBS_* represents the permeation of PBS, while *ρ_W_* and *ρ_cpa_* denote the concentrations of water and CPAs at 4 °C, respectively. *MWt_CPA_* refer to relative molecular mass of CPA. *n_CPA_*, *V_CPA_*, *Wt_CPA_*, and *C_CPA_* correspond to the number of moles, volume, weight, and concentration of CPAs that permeated the skin. *W*_1_ and *W*_2_ denote the dry weight and moist weight of the skin after immersion in the CPA.

### ESP and interaction energy simulation

Density functional theory (DFT), renowned for its superior predictive prowess, is a widely adopted computational technique in the field of chemistry. The Gaussian quantum chemistry software serves as the platform for executing DFT calculations, wherein all CPA molecules are preoptimized at the B3LYP/TZVP level before analysis.

MD simulations were conducted with Gromacs-2019.6 to investigate the interactions between solute molecules (CPAs) and water/ice in an aqueous solution system. The amber99sb-ildn field with restrained electrostatic potential atomic charge and TIP4P/Ice model were used for CPAs and water molecules. The system consisted of 74 free CPAs, 11,811 water molecules, and 1,152 ice molecules, enclosed within a simulation box measuring 59.01 × 81.21 × 127.87 Å^3^. To account for long-range electrostatic interactions, the particle-mesh Ewald method was employed with a cutoff of 1.4 nm, while the van der Waals interactions were truncated at the same cutoff. The particle-mesh Ewald method with the cutoff of 1.4 nm was used to describe the long-range electrostatic interaction by Eq. 5, while the van der Waals interaction used the same cutoff by Eq. 6. A 200-ns simulation was conducted to examine the interaction energy between CPAs and water. Besides, the ESP of CPAs was evaluated by VMD 1.9.1 and Multiwfn 3.8 [81–84].

$$E_{\mathrm{LJ}}\left(r_{\mathrm{ij}}\right)=4\varepsilon_{\mathrm{ij}}\left({\left(\frac{\varepsilon_{\mathrm{ij}}}{r_{\mathrm{ij}}}\right)}^{12}-{\left(\frac{\varepsilon_{\mathrm{ij}}}{r_{\mathrm{ij}}}\right)}^{6}\right)$$ (5)

$$E_{c}\left(r_{\mathrm{ij}}\right)=\frac{q_{i}q_{j}}{4\pi \varepsilon_{o}\varepsilon_{r}r_{\mathrm{ij}}}$$ (6)

### Low-temperature tensile tests

For low-temperature tensile tests, a low-temperature environmental chamber connected to a liquid nitrogen tank was utilized to create a freezing environment. Once the temperature reached −70 °C (the lower threshold capability of the machine), the sample was securely fixed with clamps. The stress on the sample was continuously recorded as the sample temperature varied from 25 to −70 °C (maintained for 10 min). Under the same stress test conditions, the stress–strain curve, tensile strength, fracture strain, and Young’s modulus were obtained.

### Viability assessment

All cells, primarily fibroblasts, were isolated from Sprague-Dawley rat skin tissues using the collagenase digestion method. Briefly, the skin samples were carefully dissected to remove any fat or hair and then minced into 2-mm fragments. After immersion in 1% penicillin–streptomycin solution for 15 min, the skin fragments were digested using 0.25% trypsin inhibitor for 30 min and 0.25% type I collagenase for 2 h, sequentially. The resulting cell suspension was obtained by centrifugation and subsequently cultured at 37 °C and 5% CO_2_ for further tests. One milliliter of 1 × 10^5^ ml^−1^ cell extracted from skin tissues was suspended in Dulbecco’s modified Eagle’s medium containing CPAs and then transferred into 1.8-ml cryovials (Coring 430659). The cryovials were cooled at a controlled rate of −1 °C/min to −80 °C and subsequently transferred into liquid nitrogen for long-term storage. After cryopreserved, the cryovials were rewarming in a 37 °C water bath. Cell viability was assessed using the Live/Dead kit (Invitrogen, USA), and images were captured using an inverted microscope (Nikon, JPN).

### Statistical analysis

The temperature variation of the solution during cryopreservation and warming was repeated 3 times. The research on the antifreeze properties of the CPAs involved conducting 3 parallel experiments for ice nucleation capacity, inhibition of ice growth, and prevention of recrystallization. In the MD simulation, 3 system configurations were created to ensure the random distribution of molecules within the system. The assessment of tissue histology, protein structure, biomechanical properties, and cellular viability after skin cryopreservation involved the use of a minimum of 3 replicated biological samples. Statistical significance was assessed using *t* tests, with a predefined level of significance (*P < 0.05, **P < 0.01, ***P < 0.001, ****P < 0.0001).

## Data Availability

All data associated with this study are present in the paper or the Supplementary Materials.
