# Supplementary material for: The Inhibition of Interfacial Ice Formation and Stress Accumulation with Zwitterionic Betaine and Trehalose for High-Efficiency Skin Cryopreservation
Source: Research (Wash D C). 2024 Nov 14;7:0520. doi: 10.34133/research.0520 (PMC11561590; doi:10.34133/research.0520)
Supplement: Supplementary 1 — Supplementary Materials and Methods Figs. S1 to S21 Table S1 [file research.0520.f1.docx]

**Supplementary Materials**

**The inhibition of interfacial ice formation and stress accumulation with zwitterionic betaine and trehalose for high-efficiency skin cryopreservation**

*Xinmeng Liu**^1^, Liming Zhang^1^, Haoyue Li^1^, Jing Yang^1^, and Lei Zhang^1,2^**

*1 Department of Biochemical Engineering, Frontier Science Center for Synthetic Biology and Key Laboratory of Systems Bioengineering (MOE), School of Chemical Engineering and Technology, Tianjin University, Tianjin, 300350, China.*

*2 Haihe Laboratory of Sustainable Chemical Transformations, Tianjin, 300192, China.*

*Address correspondence to: lei_zhang@tju.edu.cn (L. Zhang)

**The PDF file includes:**

Materials and Methods

Figure S1. Schem of temperature measurement.

Figure S2 The parallel experiments of the temperature measurement during the cooling process.

Figure S3. The progress of ice nucleation and growth during rapid freezing of skin.

Figure S4. Temperature variation inside the skin and surrounding aqueous solution during cooling process.

Figure S5. Simulating temperature and stress variations in the skin during the cooling process.

Figure S6. Histology and collagen content characterization of skin.

Figure S7. Biomechanical assessment of skin with various cryopreservation periods.

Figure S8. Stress relaxation behaviors with various cryopreservation periods.

Figure S9. The impact of the rewarming process on skin cryopreservation.

Figure S10. Simulating temperature and stress variations in the skin during the rewarming process.

Figure S11. Freezing point of CPAs.

Figure S12. Ice nucleation temperature of CPAs.

Figure S13. Microscopic images of ice growth rates.

Figure S14. Ice growth behaviors.

Figure S15. The thermal expansion of CPAs.

Figure S16. Mechanical properties of CPAs at cryogenic temperature conditions.

Figure S17. ESP distribution.

Figure S18. Permeation kinetics.

Figure S19. Biomechanical properties.

Figure S20. Stress-strain curve.

Figure S21. Stress relaxation behaviors.

Table S1. A summary of some input parameters to the thermal and stress models.

*Chemicals***:** Betaine (>99%) and type I collagenase was purchased from Sigma-Aldrich. Trehalose was purchased from Adamas. DMSO and Gly, Dulbecco's Modified Eagle Medium (DMEM) and fetal bovine serum (FBS) was obtained from Gibco. Penicillin-streptomycin (PS), trypsin-EDTA (0.025–0.01%), and phosphate buffered saline (PBS) Beijing Solarbio Science and Technology Co. Ltd. The culture medium for cell (keratinocytes and fibroblasts) from skin contained DMEM with 10% FBS and 1% PS. Milli-Q water (18.2 MΩ cm−1) was used in all experiments.

*Rats skin procurement***:** Prior to the surgery, the rats were anesthetized with 1.5% isoflurane and 1 L/min oxygen via inhalation. To ensure proper anesthesia, a toe pinch test was performed prior to cervical dislocation, which was used to sacrifice the rats. The dorsal skin of the rats was shaved and hair was removed using a depilatory cream (Veet, Reckitt Benckiser) before disinfecting with 75% ethanol. The entire dorsal skin was then obtained using surgical scissors and the subcutaneous tissue was removed with tissue scissors. Afterward, the ex-skin was immersed in a physiological saline solution to eliminate any residual blood.

*Thermal distribution during cooling and rewarming progress***:** To evaluate the temperature variations during the freezing and rewarming processes, we employed a 3 mm fine temperature probe and sensor (Kaipusen, China) to conduct measurements on the skin. To ensure optimal contact between the temperature sensor probe and the skin, a fixture was inserted between the rigid metal wire and the stick to provide support (Fig. S1). The stick was used to suspend the skin sample in the center of the solution. Following multiple times of cooling and rewarming to maintain consistent skin contact, temperature tests were conducted. The skin samples with 1.5 cm length, 1.5 cm width and 2 mm thickness were placed into the center of cylindrical container (diameter=2 cm) filled with water as follows. The temperature probes were strategically positioned in two distinct locations within the container: immersed in the aqueous solution in direct contact with the skin (Point 1), and located away from the skin at a distance from the container’s center equidistant with the Point 1 (Point 2).

*Thermal and stress distribution simulation details*: The water, ice and skin used were sourced from the built-in material library of the COMSOL Multiphysics software, with parameters derived semi-empirically as functions [1-5]. Specific data are shown in Table 1.

Cooling: To simulate the cooling progress, a formulation was employed to depict the time-temperature variation as follows. This involved cooling the temperature at a controlled rate of -1 °C/min until it reached -80°C, after which cryopreservation was carried out in liquid nitrogen. The system starts with an initial temperature of 4°C. The boundary temperature is dependent on T(t) (K), and cooling is applied inward until the temperature reaches the temperature of liquid nitrogen.

| $T\left( t \right)=\left\{ \begin{aligned} 277 \\ -0.017t+279.04 \\ 77 \end{aligned} \right.$ | $0\leq t\leq120 s$  $120\leq t\leq5160 s$  $5160\leq t\leq6000 s$ | (1) |
| --- | --- | --- |

Rewarming: In order to simulate the process of rewarming in a water bath, the system is initialized with a temperature of -196°C. The boundary temperature is set at 37°C. The fluid and solid heat transfer modules are coupled with the solid mechanics module, enabling a comprehensive analysis of the heat transfer process for a duration of 1000 s with the consideration of thermal expansion effects.

To discretize the geometry model for both cooling and rewarming processes, a triangular meshing module was utilized. A total of 722 triangular grids were generated with a well-formed mesh, where a finer mesh was applied at the turning points while a coarser mesh was employed elsewhere. The mesh quality was ensured with a skewness of 0.99. Throughout the investigation, the system underwent intricate phase transitions, underwent temperature fluctuations, and encountered dynamic stress variations. Rigorous data collection and analysis were conducted to scrutinize the temperature and stress profiles at distinct spatial locations within the system.

*Ice nucleation and growth test:* To observe the freezing process of skin and monitor ice nucleation and growth, 6 μm sections of fresh skin tissue were obtained using a Leica CM 1950 cryostat. These sections were placed in glass crucibles with a thin layer of surrounding water and placed in the sample room of a cryostage (LinKam, UK) with a cooling rate of 5 °C/min. Continuous recording of the ice crystal growth process was achieved using a high-speed camera-equipped microscope (Revealer, China).

*Nanoliter osmometer test:* In order to explore the ice growth capabilities of different CPAs, the growth rate of individual ice crystals was examined using an Otago nanoliter osmometer (YASN, China). In brief, 50 nL of CPAs (20 mg/mL, dissolved in water) were injected into a temperature-controlled sample holder containing silicone oil. The temperature was gradually reduced until it reached -25°C and CPAs was completely frozen. Subsequently, the holder was slowly heated until a single ice crystal appeared, with its size remaining stable. This temperature was considered the melting temperature (T_m_). A microscope (Nikon, JPN) was utilized to observe the growth of ice crystals at a specific degree of supercooling (ΔT=0.04) below T_m_.

*Ice recrystallization inhibition (IRI) test:* Ice recrystallization inhibition test was performed by splat cooling method. A 12 µL of 20 mg/mL CPA (dissolved in PBS) was dispensed from a height of 1 meter onto a glass crucible, which is then placed on a stainless steel plate in contact with liquid nitrogen vapor. The droplet spreads out and rapidly freezes on the surface of the plate. The glass crucible was moved into the sample room of cryostage and maintained at -6°C under N_2_ for 30 minutes. The cryomicrograph were captured using polarizing microscope (Nikon, Ci-POL). Evaluate the mean largest grains size (MLGS) of a maximum of ten ice crystals located within the same area and compared to a negative control of PBS.

*Differential scanning calorimetry (DSC) test:* To evaluate the formation of ice, we conducted tests on various CPAs using DSC. For each test, 20 μL of the CPAs (20 mg/mL, dissolved in water) were weighed and placed in aluminum pans, which were then analyzed using a DSC System (NETZSCH, GER). The cooling rate was set to 10 °C/min, and heat flow and temperature were recorded over the range of 20°C to -40°C. The heating rate was 2 °C/min until the temperature reached 20°C. The melting of ice was indicated by the occurrence of a prominent endothermic peak. The onset temperatures of the heat flow curves of the different CPAs were used to obtain the freezing points. We then calculated the non-freezing water contents ($W_{nf}$) of these CPAs using the following equations [6-8].

| $\left\{ \begin{aligned} W_{tc}=m_{w}/m \\ W_{f}={\Delta H/\Delta H}_{w} \\ W_{nf}=w_{tc}-w_{f} \end{aligned} \right.$ | (2) |
| --- | --- |

Where the total water content ($W_{tc}$) and freezing water content ($W_{f}$) are represented by the mass of water ($m_{w}$) and the total mass of each CPA ($m$), respectively. The melting enthalpy of CPAs ($\Delta H$) was obtained by integrating the heat flow curves from the start to the end temperature, and ${\Delta H}_{w}$ is the specific heat of water fusion, set at a constant value of 334 J/g.

*Thermal expansion test:* The thermal expansion of skin with the immersion of CPAs was assessed by TMA (NETZSCH, GER). The temperature program decreased from 20°C to -150°C, and then increased at a warming rate of 10°C/min until reaching 20°C. The thermal strain ($\varepsilon\left( T \right)$) and the thermal expansion coefficients ($\alpha\left( T \right)$) of skin sample were calculated as follows:

| $\left\{ \begin{aligned} \varepsilon\left( T \right)=\frac{L\left( T \right)-L_{0}}{L_{0}} \\ \alpha\left( T \right)=\frac{d\varepsilon\left( T \right)}{dT} \end{aligned} \right.$ | (3) |
| --- | --- |

Where $L\left( T \right)$ and $L_{0}$ were denoted the length of sample at the temperature of T and original length of the sample, respectively.

*The permeation kinetics of CPAs in skin:* In order to determine the optimal loading time of CPAs, a study was carried out on the kinetics of skin permeation. The objective was to achieve equilibrium permeation of the CPA from the skin into PBS solution. The permeation of the surrounding solution adjacent to the skin was measured using a freezing point osmometer (Tianda Tianfa, China), enabling the evaluation of CPA penetration into the skin. The specific calculation method employed is as follows ^77, 78^:

| $\left\{ \begin{aligned} n_{CPA}=\left( \pi_{CPA}-\pi_{PBS} \right)*V_{PBS}*\rho_{W} \\ {Wt}_{CPA}=n_{CPA}*{MW}_{cpa} \\ {V_{CPA}=Wt}_{CPA}/\rho_{cpa} \\ C_{CPA}=\frac{n_{CPA}}{V_{CPA}+\frac{\left( W_{2}-W_{1}-{Wt}_{CPA} \right)}{\rho}} \end{aligned} \right.$ | (4) |
| --- | --- |

Following immersion in CPA for varying durations (ranging from 1 to 180 min) at 4°C, the skin, along with the immersed CPA, was subsequently placed in PBS until osmotic pressure equilibrium was achieved ($\pi_{CPA}$). Any CPA residue left within the skin was not taken into account. $\pi_{PBS}$ represents the permeation of PBS, while $\rho_{W}$ and $\rho_{cpa}$ denote the concentrations of water and CPAs at 4°C, respectively. ${MWt}_{CPA}$ refer to relative molecular mass of CPA. $n_{CPA}$, $V_{CPA}$, ${Wt}_{CPA}$, and $C_{CPA}$ correspond to the number of moles, volume, weight, and concentration of CPAs that permeated the skin. $W_{1}$ and $W_{2}$ denote the dry weight and moist weight of the skin after immersion in the CPA.

*Collagen assessment:* Cryopreserved skin samples were evaluated by a HYP detection kit (Solarbio, China), with fresh skin as the positive control. The experimental procedures followed the instructions provided, and the results were calculated using the formula provided by the kit.

*Biomechanical tests:* The biomechanical properties of cryopreserved skin samples were evaluated using an electronic universal testing machine (SUNS, China) equipped with a 500 N load cell, with fresh skin serving as the positive control. Dumbbell-shaped samples were prepared with dimensions of 10 mm gauge length and 4 mm width. The samples were subjected to a loading rate of 10 mm/min in room temperature. Tensile strength, fracture strain, and Young's modulus (the slope of the linear region in the stress-strain curves) were determined from the stress-strain curves. Additionally, stress relaxation properties were assessed by applying 25% strain and 100% strain to the samples, allowing them to relax for 10 minutes. The stress remaining in the samples was calculated by dividing the stress at the end of the relaxation period by the maximum stress. The final stress relaxation was determined by calculating the average stress during the last 50 seconds.

*Histology:* After cryopreservation and rewarming, the skin samples with different CPAs were clipped and fixed in 4% paraformaldehyde for 4 h. after fixed, the dehydrated in 40% sucrose solution for 12 h and optimal cutting temperature (OCT) compound were conducted respectively. The 6 um sections were cryosectioned by a Leica CM 1950 cryostat and stained with H&E staining kit (Solarbio, China) and picrosirius red staining kit (Solarbio, China). Skin tissue density quantification was assessed by comparing the area of the frozen skin to that of the fresh skin.


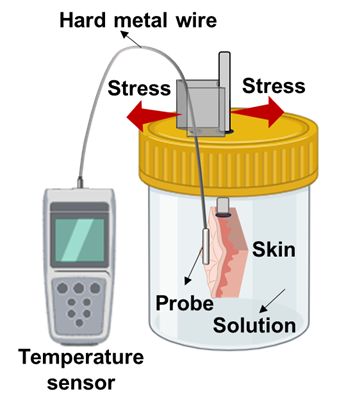


**Figure S1.** Schem of temperature measurement.

**
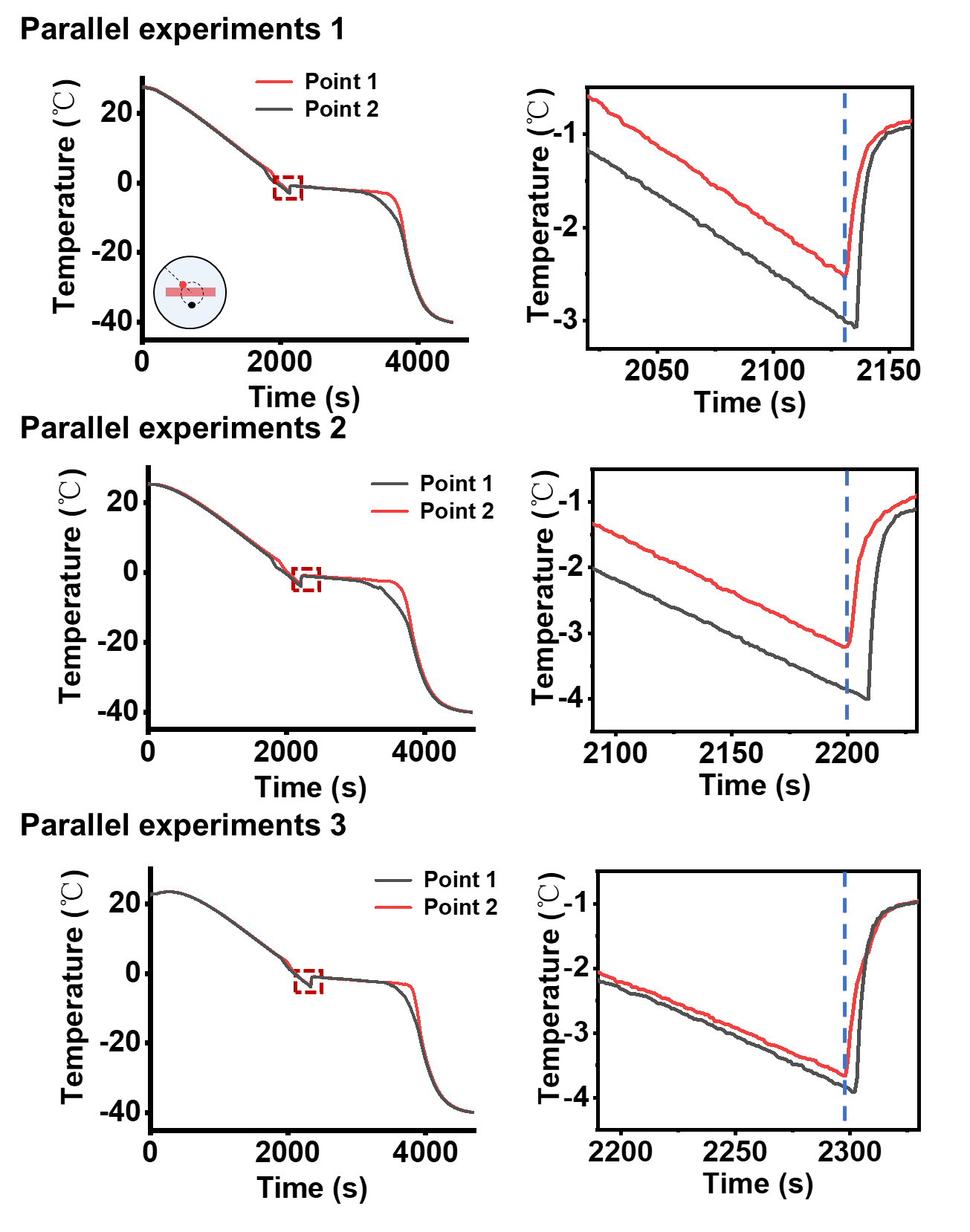
**

**Figure S2** The parallel experiments of the temperature measurement during the cooling process.


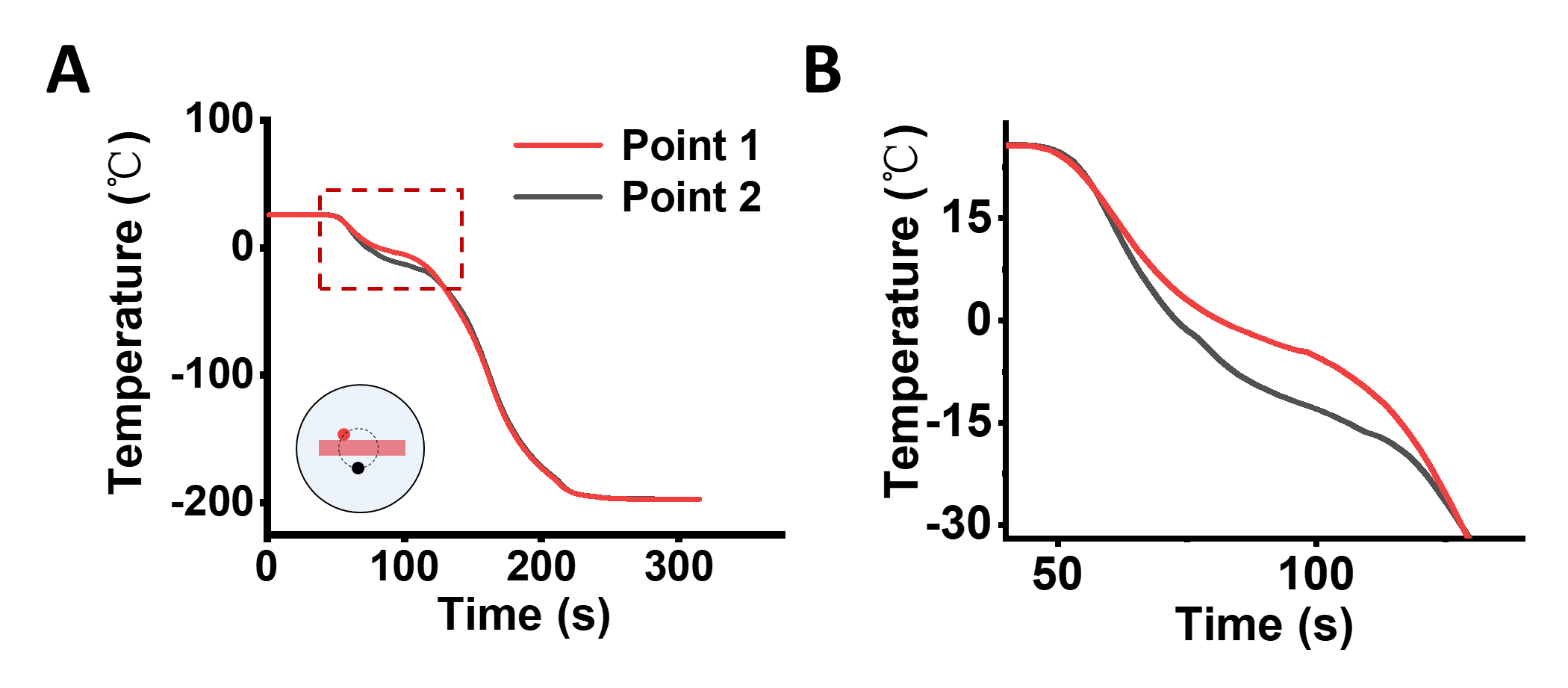


**Figure S3.** The progress of ice nucleation and growth during rapid freezing of skin. (A) Temporal variation of temperature with (B) an enlarged detailed view during skin freezing (plunging the skin into LN2).


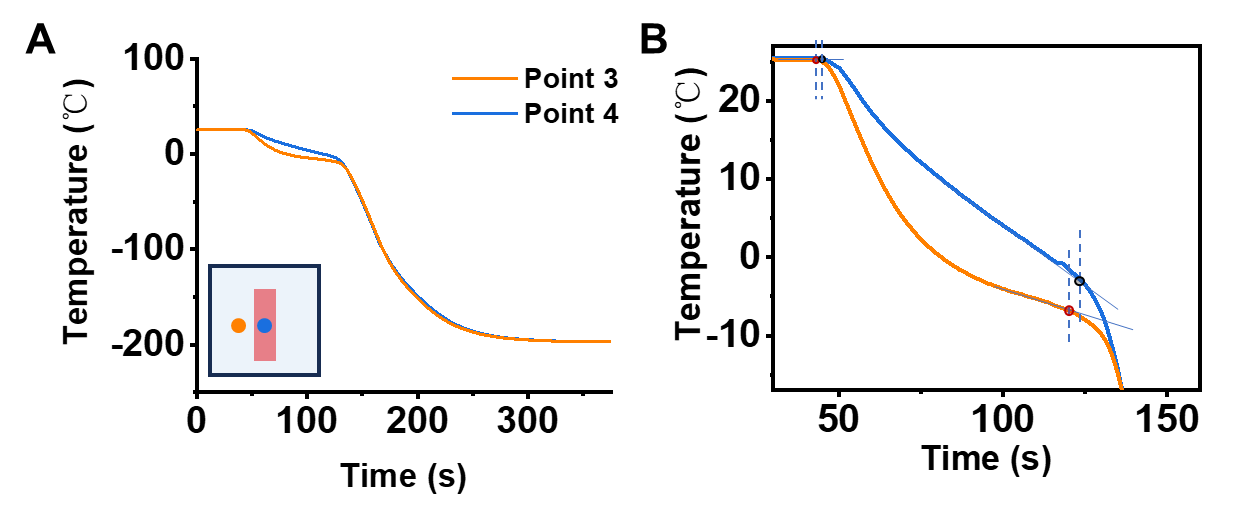


**Figure S4.** Temperature variation inside the skin and surrounding aqueous solution during cooling process. The temperature probs were (A) embedded inside the skin (Point 3) and in surrounding solution (Point 4) with (B) an enlarged detailed view during the cooling process.


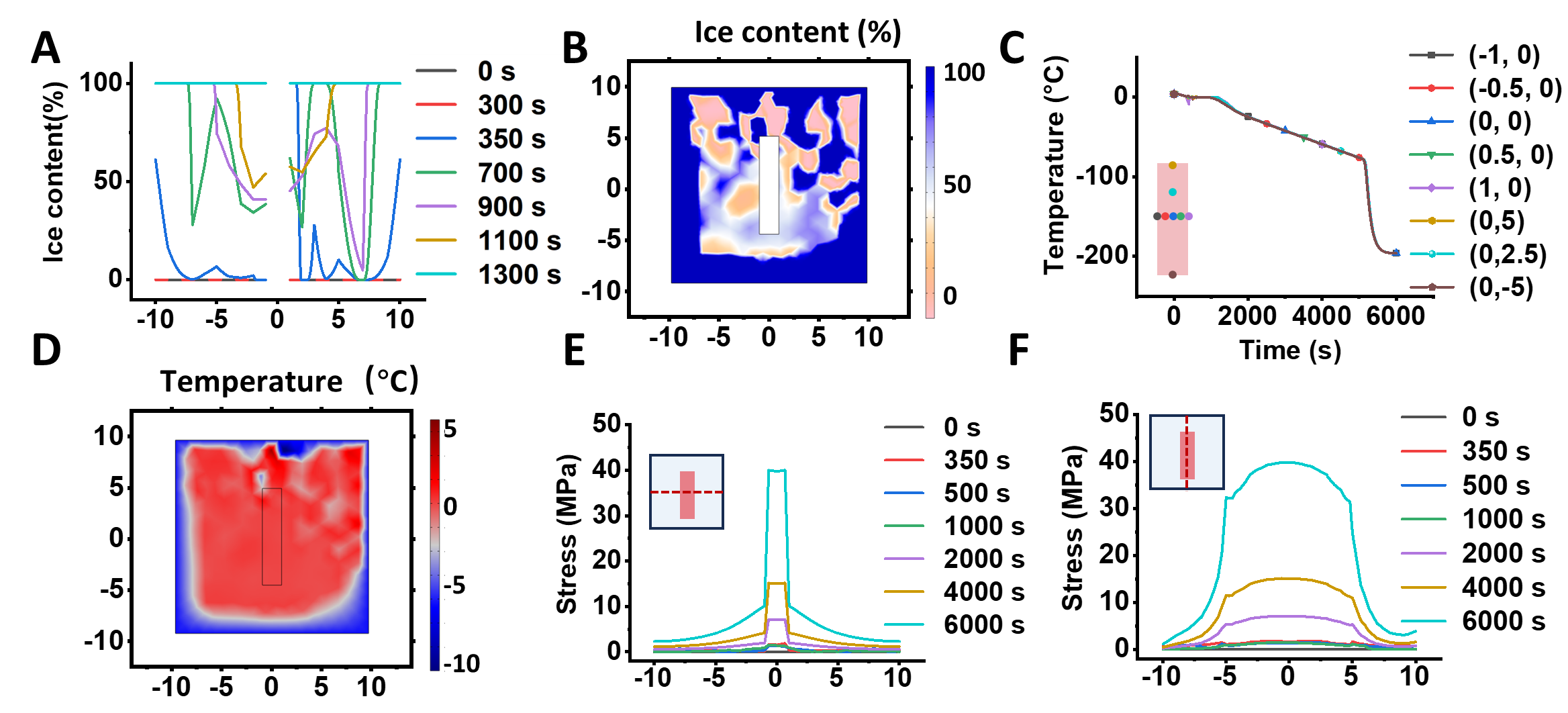


**Figure S5.** Simulating temperature and stress variations in the skin during the freezing process. (A) Ice content distribution the skin inner. (B) Ice content at the time of 700 s. (C) Temperature distribution the skin inner. (D) Temperature distribution at the same time of 700 s. (E) Horizontal and (F) vertical stress distribution under various time intervals.


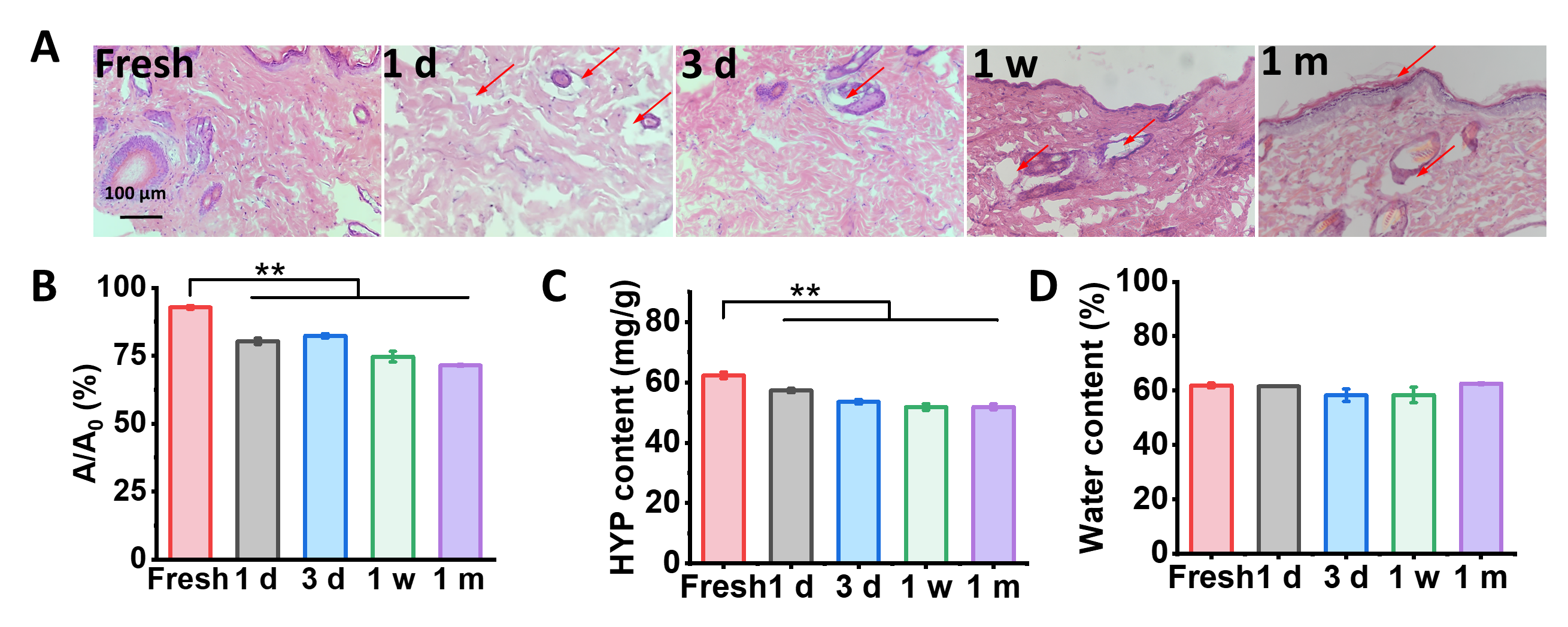


**Figure S6.** Histology and collagen content characterization of skin with various cryopreservation periods. (A) Micrographs of cryopreserved skin without CPAs stained with H&E (purple: chromatin within the cell nucleus and nucleic acids; eosinophilic pink: the cytoplasm and extracellular matrix). (B) Skin tissue density quantification. (C) HYP content. (D) Water content of skin.


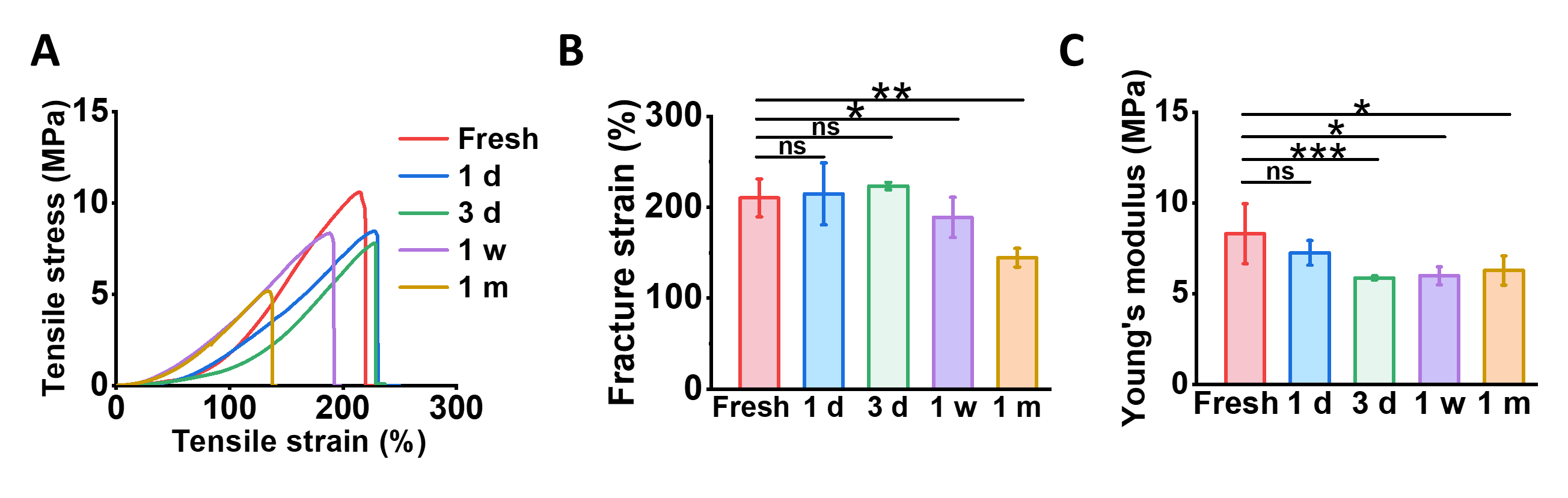


**Figure S7.** Biomechanical assessment of skin without CPAs for various cryopreservation periods. (A)The stress-strain curve. (B) Fracture strain. (C) Young’s modulus. The cryopreservation periods were 1 day, 3 days, 1 week and 1 month, respectively.


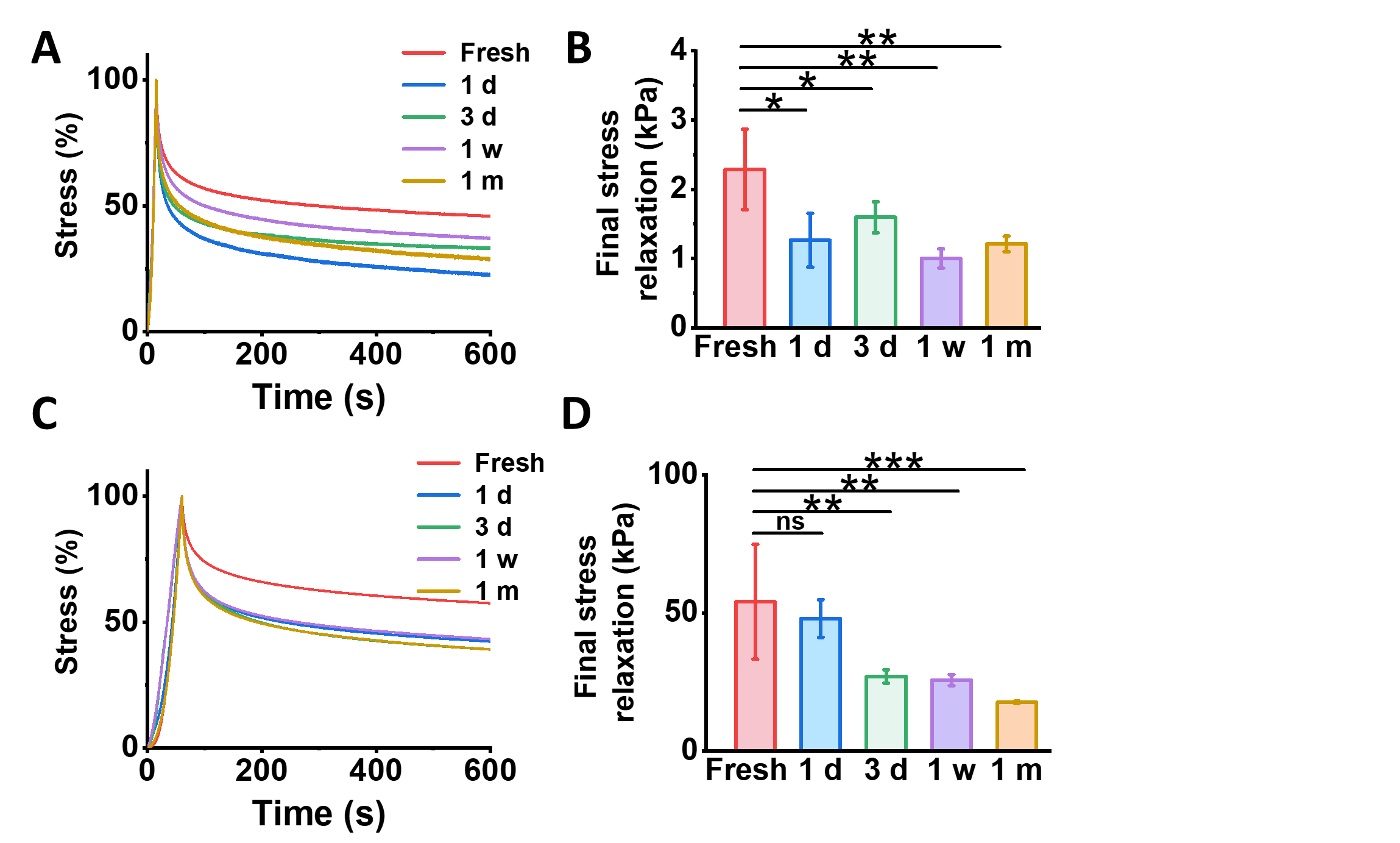


**Figure S8.** Stress relaxation behaviors of skin without CPAs for various cryopreservation periods. (A) Strain relaxion carve, and (B) final stress remain were analyzed at 25% strain. (C) Strain relaxion carve, and (D) final stress remain were analyzed at 100% strain. The cryopreservation periods were 1 day, 3 days, 1 week and 1 month, respectively.


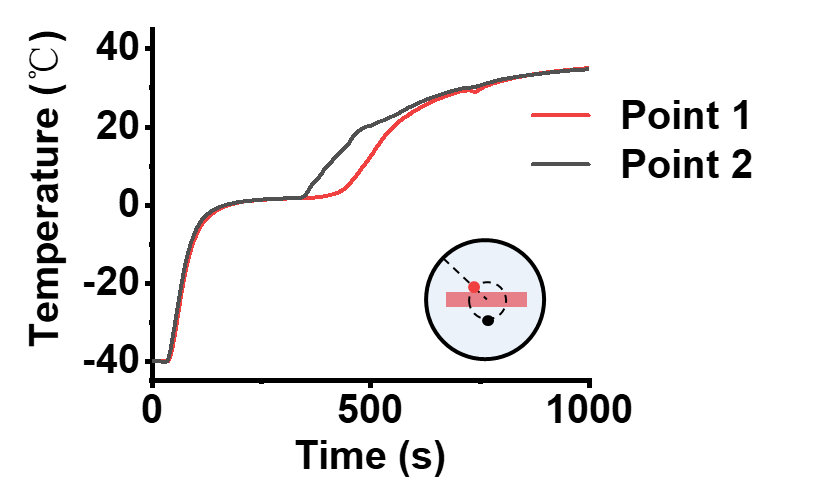


**Figure S9.** The impact of the rewarming process on skin cryopreservation. Temperature variation of the skin in contact (Point 1) and the skin situated at a certain distance (Point 2, same concentric circles of Point 1).


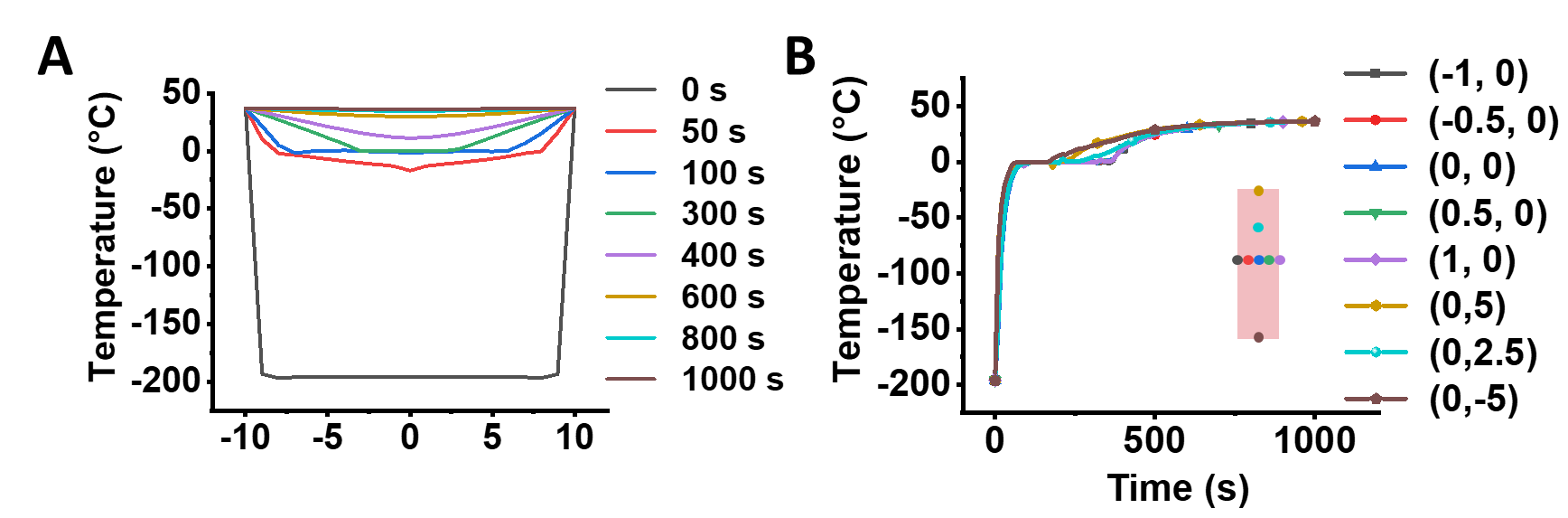


**Figure S10.** Simulating temperature and stress variations in the skin during the rewarming process. Temperature distribution for different (A) time points and (B) locations.


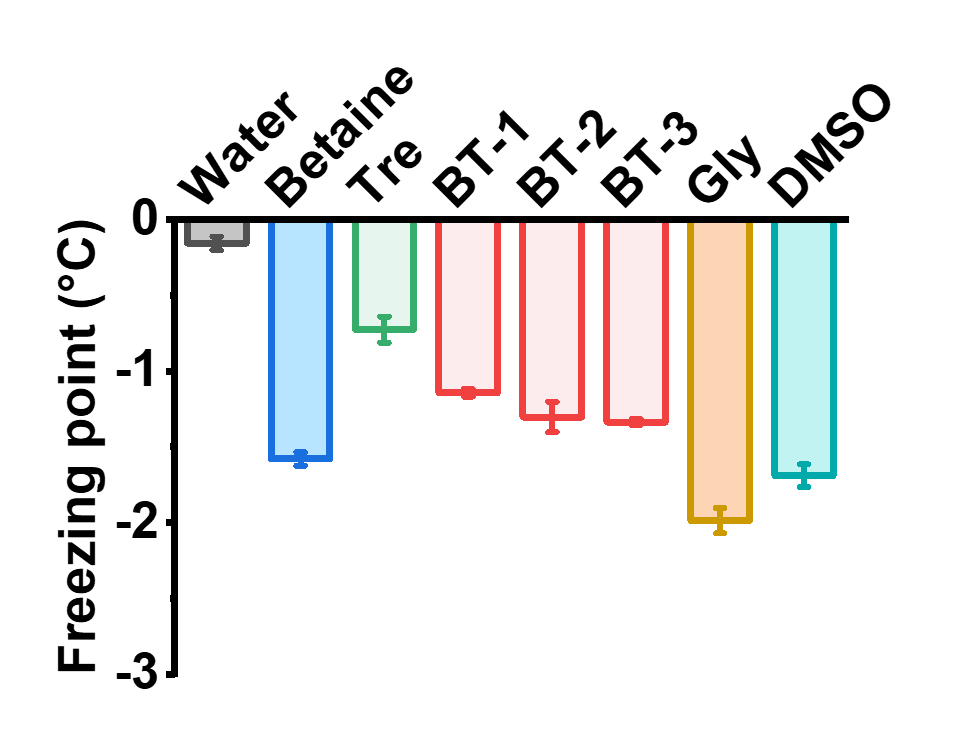


**Figure S11.** Freezing point of CPAs. The freezing point of different CPAs with a concentration of 15%wt content were tested by DSC.

**
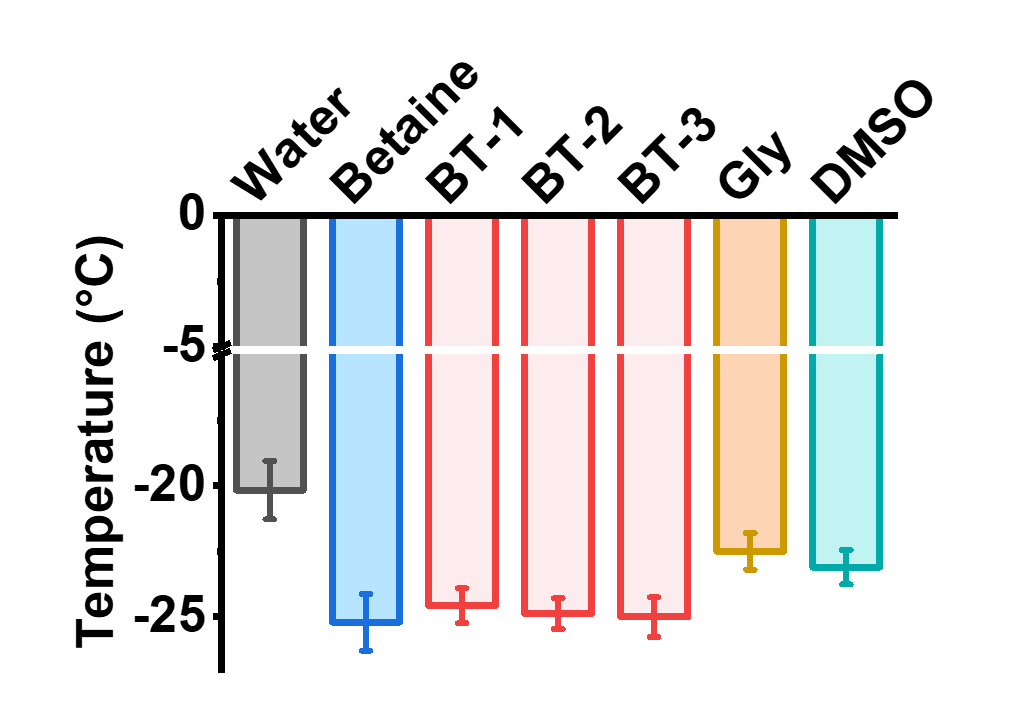
**

**Figure S12.** Ice nucleation temperature of CPAs. The freezing point of different CPAs with a concentration of 15%wt content were tested by optical microscopy**.**

**
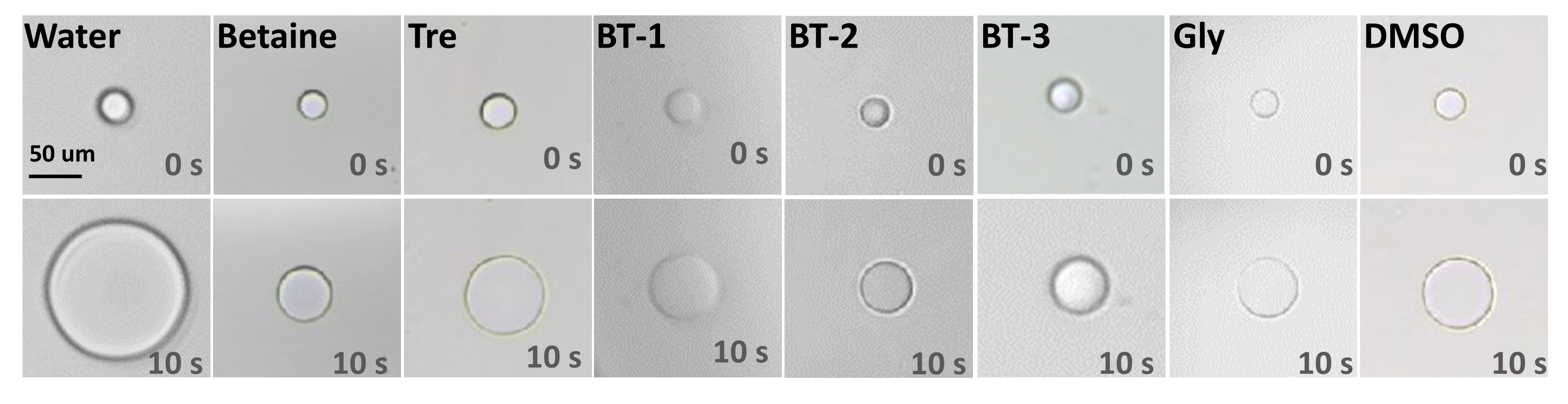
**

**Figure S13.** Microscopic images of ice growth rates. The single ice with CPAs were recorded in 0 s and 10 s.


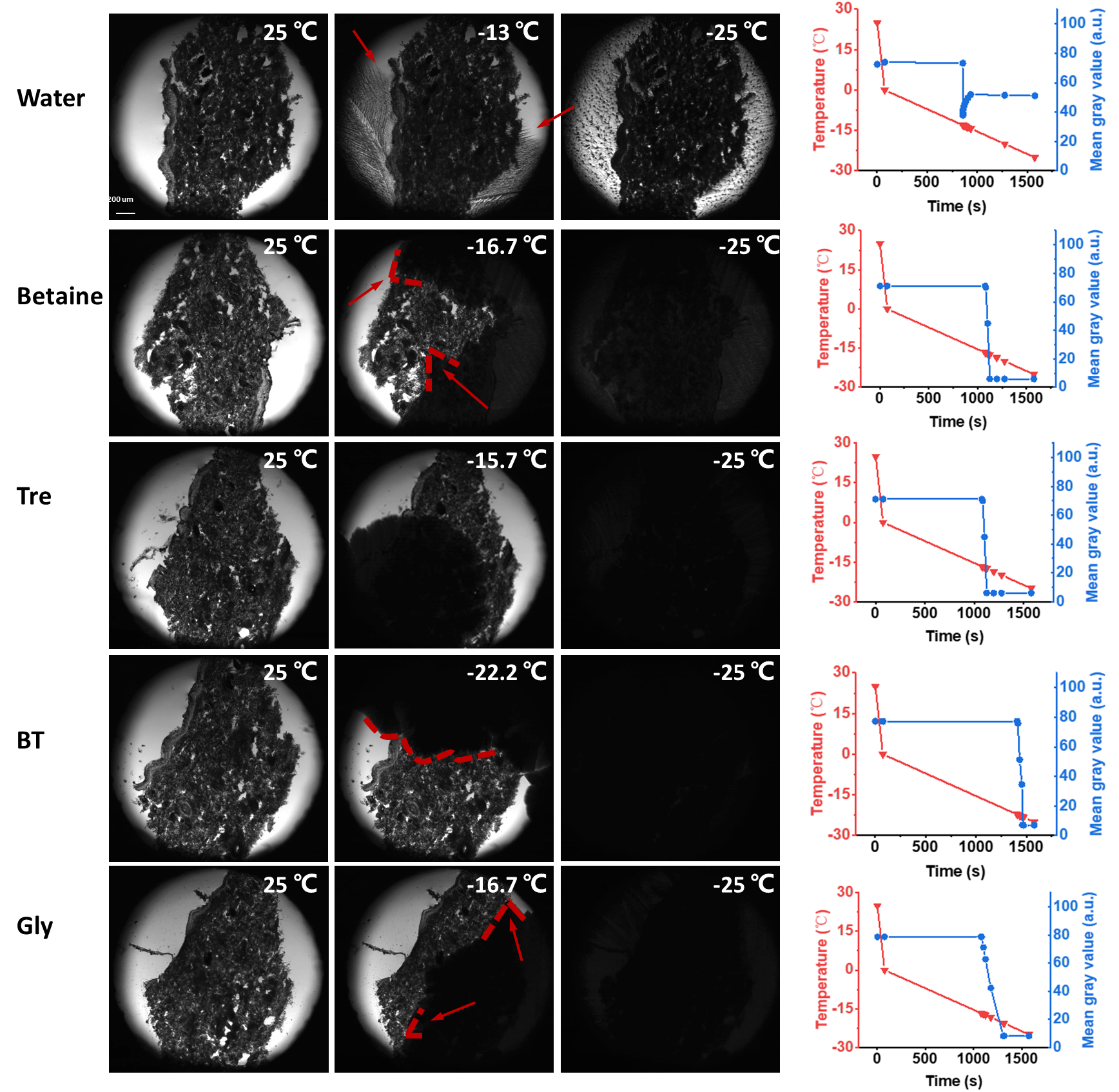


**Figure S14.** Ice growth behaviors. Cryomicrographs and quantitative assessment of the ice crystal growth during skin cryopreservation using different CPAs (15wt%). The arrow: the sharp ice front.


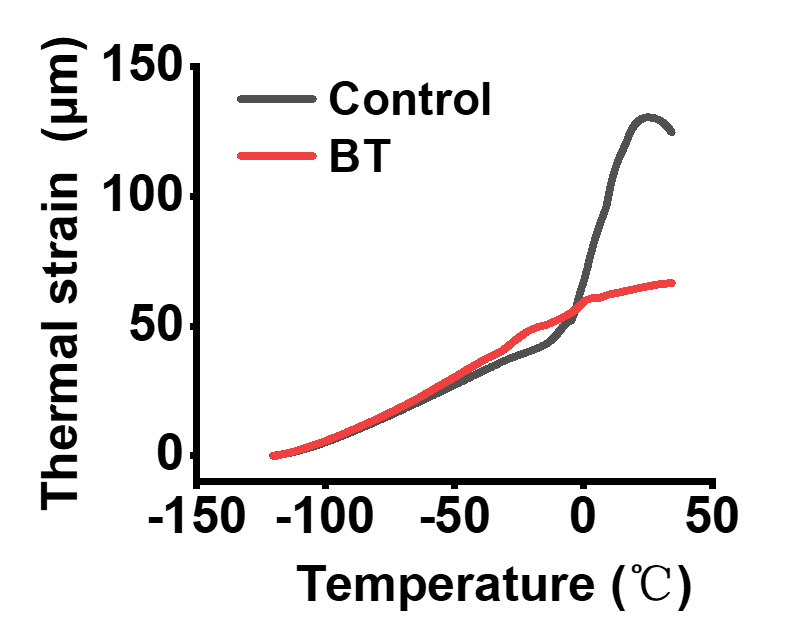


**Figure S15.** The thermal expansion of CPAs. Thermal expansion curve of skin with BT and without CPAs (C group).


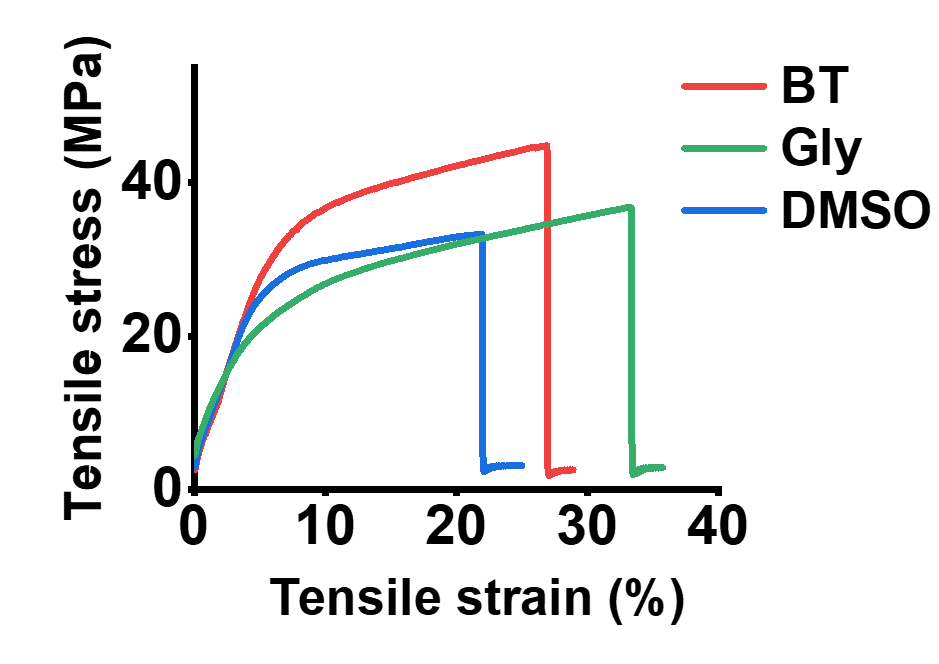


**Figure S16.** Mechanical properties of CPAs at cryogenic temperature conditions. The stress-strain curve of CPAs at the temperature of 70 °C.


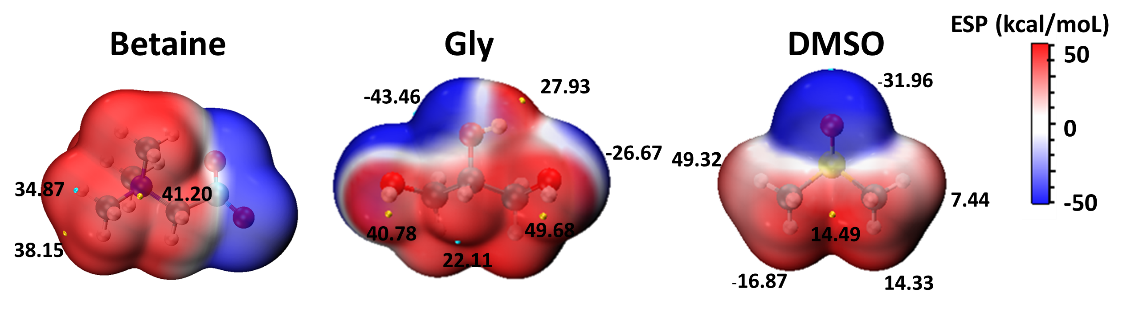


**Figure S17.** ESP distribution. ESP distribution maps of B, G and D in the opposite direction. Blue ball: local maximum; Yellow ball: local minimum.


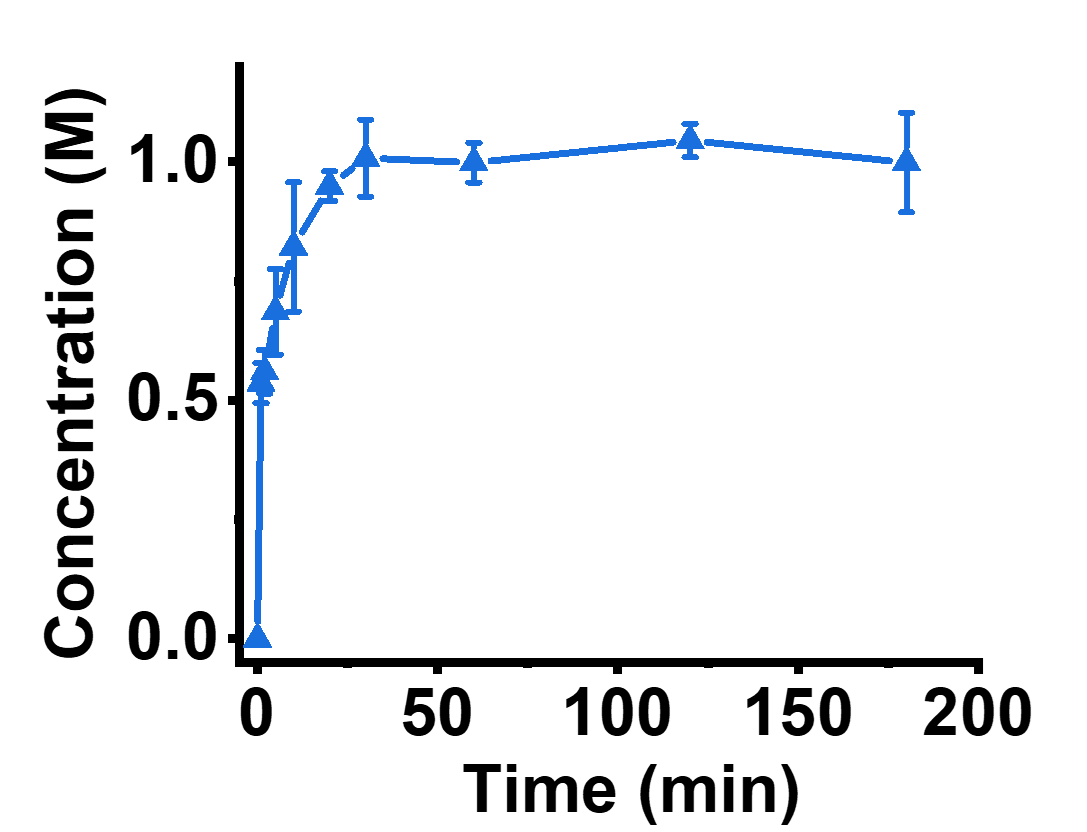


**Figure S18.** Permeation kinetics. Permeation of 1.06 M BT (15wt%) into rat skin at 4◦C over 24 h.


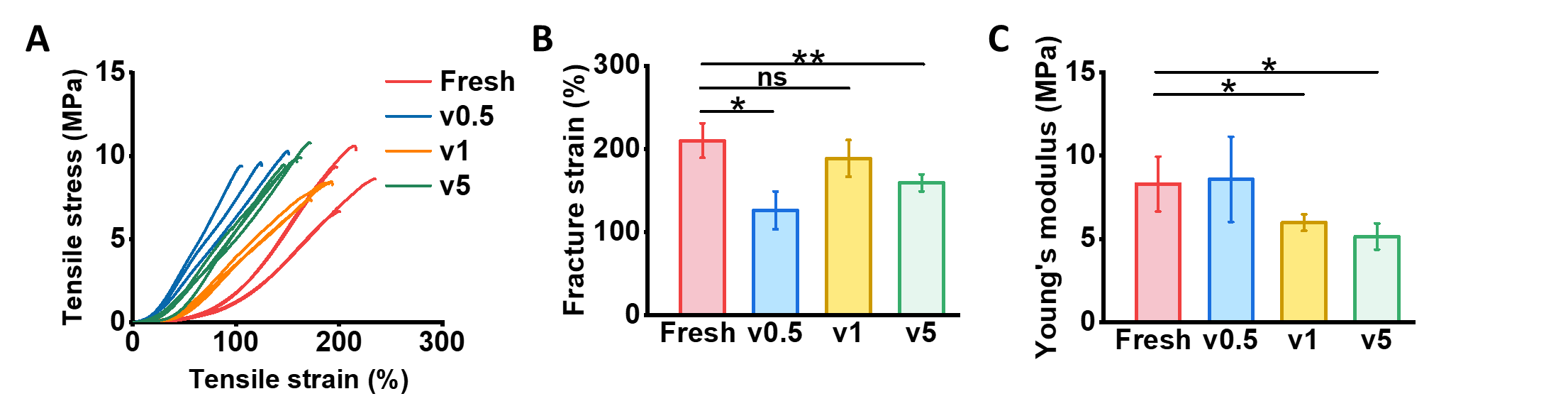


**Figure S19.** Biomechanical properties. (A) Stress-strain curve, (B) Fracture strain, and (B) Young’s modulus were analyzed by different gradient freezing rate, including 0.5, 1, 5 mm/min.


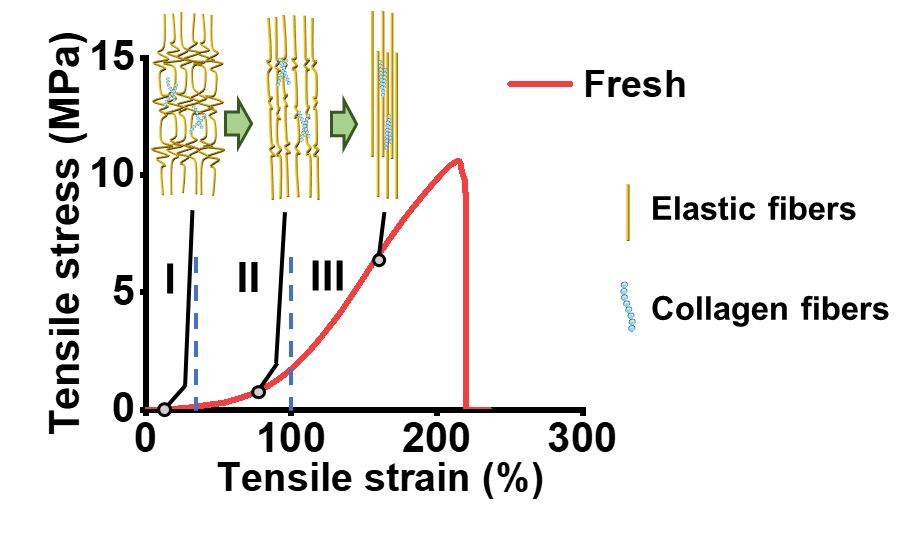


**Figure S20.** Stress-strain curve. The stress-strain curve of skin displayed "J" shape and three regions, including I-toe region, II-heel region and III-linear region.


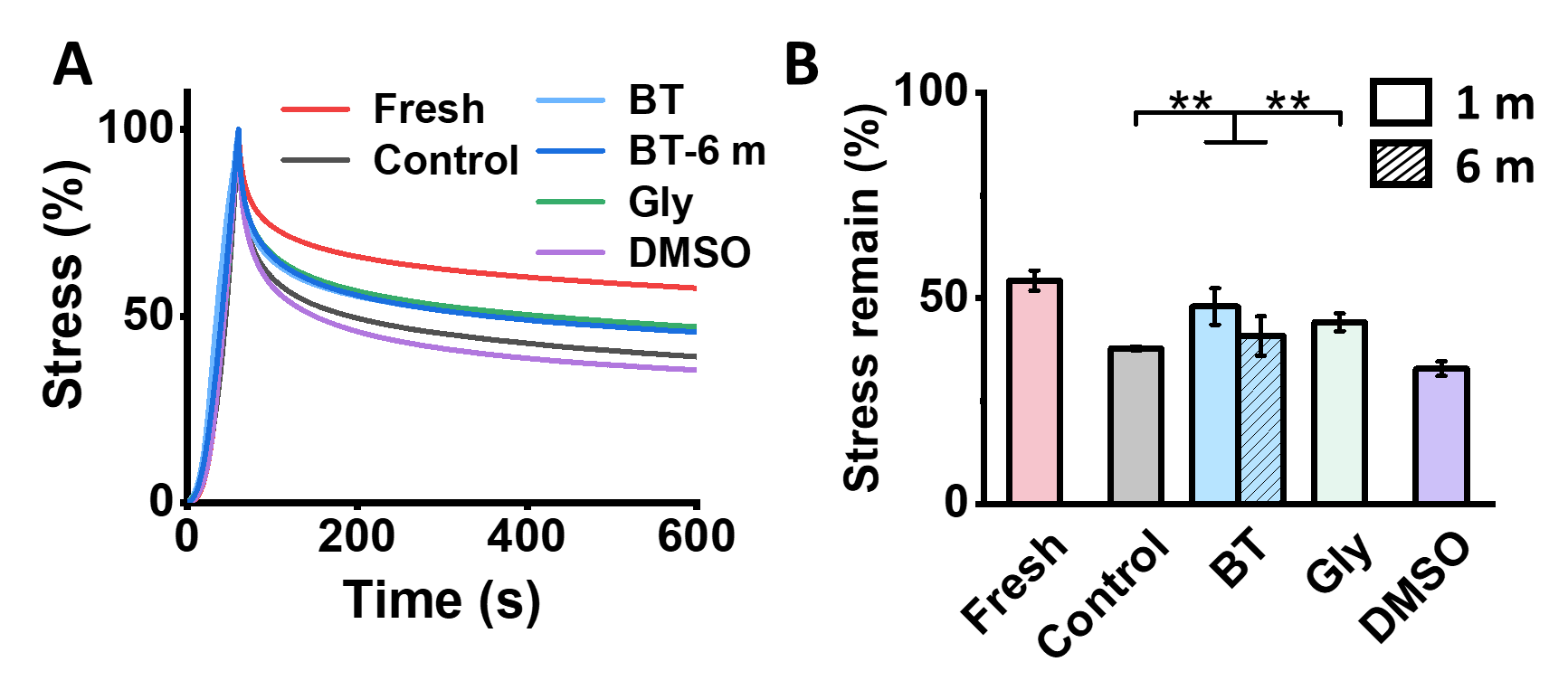


**Figure S21.** Stress relaxation behaviors. When 100% strain, (A) Strain relaxion carve, and (B) final stress remain were analyzed.

**Table S1** A summary of some input parameters to the thermal and stress models

|  | Property | Value | Units |
| --- | --- | --- | --- |
| Skin | Thermal expansion coefficient, α_skin_ | 6×10^-5^ | 1/K |
|  | Young’s module, E _skin_ | 10 | GPa |
|  | Poisson’s ratio, ε_skin_ | 0.33 | 1 |
|  | Thermal conductivity of unfrozen skin, k_skin-1_ | 0.5 | W/(m·K) |
|  | Thermal conductivity of frozen skin, k_skin-2_ | 2 | W/(m·K) |
|  | Density, ρ_skin_ | 1109 | kg/m³ |
| Water | Thermal conductivity, k_water_(T) | -0.87+0.009T-1.58×10^-5^T^2^+7.98×10^-9^T^3^ (273<T<293) | W/(m·K) |
|  | Density, ρ_water_(T) | -950.70+18.92T-0.060T^2^+0.000063T^3^ (273<T<293) | kg/m³ |
| Ice | Thermal conductivity, k_ice_(T) | 48.48-1.44T+0.020T^2^-1.38×10^-4^T^3^+4.75×10^-7^T^4^-6.44×10^-10^T^5^ （73<T<190）；  9.82-0.049T+7.60×10^-5^T^2^ （190<T<273） | W/(m·K) |
|  | Thermal expansion coefficient, α_ice_(T) | 2.27×10-5+1.24×10^-7^T (73<T<273) | 1/K |
|  | Density, ρ_ice_(T) | 933.7+0.037×T-3.87×10^-4^T^2^+8.84×10^-8^T^3^ (73<T<273) | kg/m³ |

**Reference:**

[1] He X, Bischof JC, Analysis of thermal stress in cryosurgery of kidneys. *J. Biomech. Eng.* 2005;127(4):656-61.

[2] Hoffmann NE, Bischof JC, Cryosurgery of normal and tumor tissue in the dorsal skin flap chamber: Part I--thermal response. *J. Biomech. Eng.* 2001;123(4):301-9.

[3] Xu F, Seffen KA, Lu TJ, Non-Fourier analysis of skin biothermomechanics. *International Journal of Heat and Mass Transfer*. 2008;51(9):2237-2259.

[4] Eisenberg DP, Steif PS, Rabin Y, On the Effects of Thermal History on the Development and Relaxation of Thermo-Mechanical Stress in Cryopreservation. *Cryogenics (Guildf)*. 2014;64:86-94.

[5] Li X, Luo P, Qin QH, Tian X, The phase change thermoelastic analysis of biological tissue with variable thermal properties during cryosurgery. *Journal of Thermal Stresses*. 2020;43:1016 - 998.

[6] Qiao C, Ma X, Zhang J, Yao J, Effect of hydration on water state, glass transition dynamics and crystalline structure in chitosan films. *Carbohydr. Polym.* 2019;206:602-608.

[7] Kocherbitov V, The nature of nonfreezing water in carbohydrate polymers. *Carbohydr. Polym.* 2016;150:353-8.

[8] Liu WG, Yao KD, What causes the unfrozen water in polymers: hydrogen bonds between water and polymer chains? *Polymer*. 2001;42(8):3943-3947.
